# Selfing Promotes Spread and Introgression of Segregation Distorters in Hermaphroditic Plants

**DOI:** 10.1093/molbev/msae132

**Published:** 2024-06-27

**Authors:** Hongru Wang, Léo Planche, Vladimir Shchur, Rasmus Nielsen

**Affiliations:** Shenzhen Branch, Guangdong Laboratory of Lingnan Modern Agriculture, Key Laboratory of Synthetic Biology, Ministry of Agriculture and Rural Affairs, Agricultural Genomics Institute at Shenzhen, Chinese Academy of Agricultural Sciences, Shenzhen, China; State Key Laboratory of Tibetan Plateau Earth System, Environment and Resources (TPESER), Institute of Tibetan Plateau Research, Chinese Academy of Sciences, Beijing 100101, China; Department of Integrative Biology, UC Berkeley, Berkeley, CA, USA; Laboratoire Interdisciplinaire des Sciences du Numérique, Université Paris Saclay, Gif-sur-Yvette, France; International laboratory of statistical and computational genomics, HSE University, Moscow 109028, Russian Federation; Department of Integrative Biology, UC Berkeley, Berkeley, CA, USA

## Abstract

Segregation distorters (SDs) are genetic elements that distort the Mendelian segregation ratio to favor their own transmission and are able to spread even when they incur fitness costs on organisms carrying them. Depending on the biology of the host organisms and the genetic architecture of the SDs, the population dynamics of SDs can be highly variable. Inbreeding is considered an effective mechanism for inhibiting the spread of SDs in populations, and can evolve as a defense mechanism against SDs in some systems. However, we show that inbreeding in the form of selfing in fact promotes the spread of SDs acting as pollen killers in a toxin–antidote system in hermaphroditic plants by two mechanisms: (i) By reducing the effective recombination rate between killer and antidote loci in the two-locus system and (ii) by increasing the proportion of SD alleles in individual flowers, rather than in the general gene-pool. We also show that in rice (*Oryza sativa* L.), a typical hermaphroditic plant, all molecularly characterized SDs associated with pollen killing were involved in population hybridization and have introgressed across different species. Paradoxically, these loci, which are associated with hybrid incompatibility and can be thought of as Bateson–Dobzhansky–Muller incompatibility loci are expected to reduce gene-flow between species, in fact cross species boundaries more frequently than random loci, and may act as important drivers of introgression.

## Introduction

One of the basic rules in biology is Mendel's law of segregation, which states that the 2 alleles at a locus are transmitted to offspring with equal probability. Segregation distorters (SDs) are alleles that violate this law and are transmitted to the offspring with larger than 50% probability (Lyttle 1991), causing segregation distortion, i.e. deviations from Mendel's law. Since they were first discovered nearly 100 years ago (Gershenson 1929), SDs are found to be ubiquitous across the tree of life, including in insects (Sandler et al. 1959; Beeman et al. 1992), fungi (Turner and Perkins 1991; Vogan et al. 2022), plants (Lu et al. 2002; Fishman and Kelly 2015), nematodes (Ben-David et al. 2021), and mammals (Silver 1985). SDs are often considered to be selfish elements (Burt and Trivers 2009), as they may spread in the population even though they may cause a fitness disadvantage to the individuals carrying them.

Segregation distortion can be caused by diverse mechanisms at both cytological and molecular levels. Specific chromosomal structures, for example, the heterochromatic knobs that preferentially make their way to female gametes, can cause transmission ratio bias during meiosis (Rhoades 1942; Longley 1945; Fishman and Saunders 2008). B chromosomes, which are supernumerary chromosomes found in many species and are often segregating in populations, can also distort transmission ratios by being preferentially retained in gametes (Jones 1991). A specific genomic locus, i.e. a genic SD, can be inherited by offspring with larger than 50% probability, often through destruction of the gametes carrying the alternative allele. Recent advances in molecular dissection of genic SD loci revealed a recurrent pattern that these SDs typically are caused by two or more genes that are closely linked (Sweigart et al. 2019; Burga et al. 2020), often consistent with the “toxin–antidote” model in which the killer haplotype carries functional copies of toxin and antidote, while sensitive haplotypes lack them or carry nonfunctional copies. Genetically, the “toxin–antidote” model involves two loci with each having two alleles (A/a and B/b), and there is incompatibility between “a” and “b” alleles. Segregation distortion occurs when the sensitive haplotype (aB) is killed by the killer haplotype (Ab). This system is similar to a Bateson–Dobzhansky–Muller model (Bateson 1909; Dobzhansky 1934; Orr 1996), which might be more familiar to some readers, if the “a” and “b” alleles have arisen in two different species.

Depending on the mechanism through which SDs gain selective advantage, SDs can be classified as either relative or absolute (Lyttle 1991; Martinossi-Allibert et al. 2021). From a heterozygous parent, relative SDs gain advantage by reducing the copy number of alternative alleles passed to progenies, while absolute SDs increase the absolute copies of themselves. Relative SDs can lead to fitness loss as they often reduce the fecundity of individuals carrying them, while absolute SDs generally do not decrease the fecundity of their hosts. Consequently, relative SDs have a much smaller selective advantage over alternative alleles in a population when compared with absolute SDs, especially when they are in low frequency (Nauta and Hoekstra 1993; Martinossi-Allibert et al. 2021).

SDs can sweep through the population and quickly go to fixation if they do not encounter opposing forces. However, the efficacy of segregation distortion is affected by many factors, including the specifics of the mating system of the hosts. Genetic modeling based on B chromosomes (Burt and Trivers 1998) and genic SDs based on the mating systems in yeast (Hernández et al. 2021; Martinossi-Allibert et al. 2021) and nematodes (Noble et al. 2021) suggest that inbreeding inhibits the spread of SDs. Moreover, by modeling the population dynamics of synthetic gene drive, Bull (2016) and Bull et al. (2019) showed that selfing, i.e. self-fertilization, or other forms of inbreeding could evolve in a hermaphroditic population as an effective way to block the spread of the drive. If inbreeding thwarts the spread of SDs, we should predict that SDs will be rare in inbreeding species. However, genetic mapping studies have shown an abundance of SDs in highly inbred plant species, including *Arabidopsis* (Simon et al. 2022), rice (Causse et al. 1994; Harushima et al. 1996; Yamagishi et al. 1996; Xu et al. 1997), barley (Graner et al. 1991; Zivy et al. 1992), pearl millet (Busso et al. 1995; Liu et al. 1996), common bean (Paredes and Gepts 1995), and *Aegilops tauschii* (Faris et al. 1998), as well as many other cultivated and wild hermaphroditic plant species. To reconcile the inconsistency between the theoretical prediction and empirical observations, we develop models based on several well-characterized toxin–antidote systems (reviewed in Sweigart et al. 2019; Burga et al. 2020) to investigate the dynamics of SDs in hermaphroditic plants. We explore how their unique mating system interacts with important factors including recombination rate, selfing rate, initial SD allele frequency, and pollen redundancy levels, to determine the spread of SDs. The level of pollen redundancy is here defined as the number of pollen grains delivered to a flower divided by the number of pollen grains needed to fertilize all ovules of the flower. We find that selfing reduces the effective recombination rate (Nordborg and Donnelly 1997; Conway et al. 1999; Nordborg 2000) between the two loci underlying an SD, and it also increases the proportion of SD alleles in individual flowers through the “pollen sequestration” effect, which are both advantageous for the spreading of segregation distortion. We also provide empirical analyses of SDs in rice, a typical hermaphroditic plant, and show that SDs frequently spread across species boundaries.

## Results

**Fig. 1.**
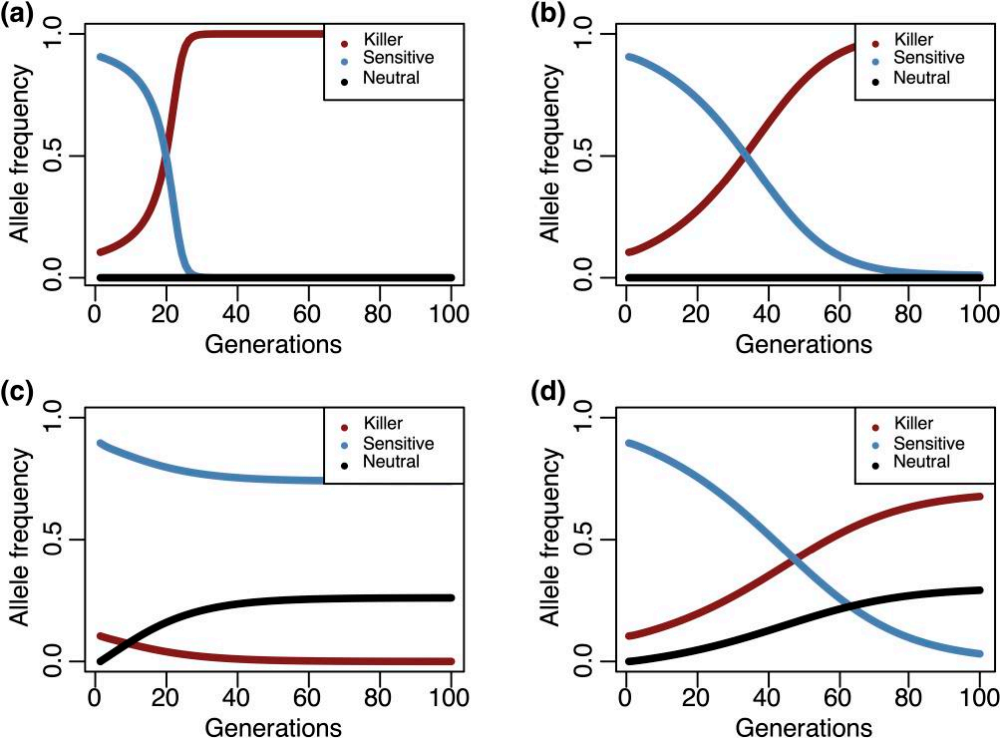
Expected allele frequency of different haplotypes as a function of time since the introduction of the SD haplotype. The calculations assume initial frequencies of 0.1 and 0.9 for the killer and the sensitive haplotypes, respectively. The pollen redundancy level, *R*, is set as 10. The killing efficiency parameter, *k*, is set as 1. In a), the simplest case, there is neither selfing nor recombination, the killer haplotype simply replaces the sensitive haplotype. The recombination rate, *r*, is set as 0 and the selfing rate, *s*, is set as 1*e*–5. In b), selfing (*s* = 0.9) is added; in c), recombination is added (*r* = 0.1), and in d), both recombination (*r* = 0.1) and selfing (*s* = 0.9) are added.

Our model assumes a hermaphroditic plant system with perfect flowers, segregation distortion affecting pollen only, and a causative two-loci, toxin–antidote genetic system (Methods). There are two alleles (A/a and B/b) at each locus and four possible haplotypes: the sensitive haplotype (aB) that can be killed by the killer haplotype (Ab); the neutral haplotype (AB) that can neither kill other haplotypes nor be killed itself; the suicide haplotype (ab) that contains incompatible alleles (see Methods for details). Furthermore, we assume that there is no other viability effect on diploid individuals associated with different haplotypes. We first explore the dynamics of a population in which the sensitive haplotype exists at high frequency (90%) and the killer haplotype is introduced at a moderate frequency (10%) (Fig. 1). Most of the relevant dynamics is exposed by considering this scenario. With full-linkage between the two loci and assuming cross-fertilization in the population, there are only two haplotypes in the population throughout the modeling (killer and sensitive), and the killer haplotype will increase in frequency and go to fixation (Fig. 1a). We also tested whether the pollen redundancy level of a flower influences the dynamics. Pollen redundancy (*R*) measures numerical redundancy of pollens, which is defined as the number of pollen grains delivered to a flower divided by the number of pollen grains needed to fertilize all ovules of the flower, where high redundancy indicates that reducing the number of pollens is less likely to cause fertility reduction of a flower (see Methods for mathematical definitions). We found that the outcome remained the same regardless of whether the pollen redundancy level is high (*R* = 10, Fig. 1a) or low (*R* = 1.5 or 1, supplementary fig. S1, Supplementary Material online) under the full-linkage and out-crossing scenario, as the fitness of different genotypes remains similar and the killer haplotype will always kill the sensitive haplotype in the heterozygous state. When the selfing rate is high (*s* = 0.9) and assuming full-linkage, the spread of the killer haplotype is thwarted as its allele frequency rises more slowly (Fig. 1b). The equilibrium state could also vary depending on the pollen redundancy level under this full-linkage, high-selfing scenario: with high redundancy, the sensitive haplotype will ultimately be replaced by the killer haplotype as long as *s* < 1; with low pollen redundancy, the heterozygotes have much lower fitness with pollen supply nearly cut to half, which will lead to elimination of the killer haplotype in the end (supplementary fig. S2, Supplementary Material online). When allowing recombination between the two loci (*r* = 0.1), other equilibrium states (Fig. 1c) can be found than those observed in the full-linkage scenario (Fig. 1a). The neutral haplotype is formed through recombination and increases in frequency quickly. Perhaps surprisingly, the killer haplotype drops in frequency, because the suicide haplotype can be formed as a recombinant from the killer haplotype, reducing the fitness of the SD allele. Under this scenario, the neutral haplotype has the highest fitness (supplementary fig. S4, Supplementary Material online), while both the sensitive and killer haplotypes decrease in frequency until the SD allele is lost. When the killer allele is lost, the fitness of the sensitive and the neutral haplotypes becomes the same and equilibrium is reached, with both the sensitive and the neutral alleles segregating in the population (although in the presence of drift one or the other could be lost). With high pollen redundancy (*R* = 10), there is a balance between the relative fitness loss due to suicide haplotype formation and the fitness gained by killing competing sensitive haplotypes for the killer haplotype. This balance is dependent on the haplotype composition of the population. The equilibrium state varies depending on the initial frequency, with either the killer or the sensitive haplotypes coexisting with the neutral haplotype (supplementary fig. S3, Supplementary Material online). When further adding selfing to the model, a different equilibrium state can be reached in which the sensitive haplotype is lost and the neutral and the killer haplotypes coexist in the population (Fig. 1d). Selfing reduces the effective recombination rate (Nordborg and Donnelly 1997; Conway et al. 1999; Nordborg 2000), leading to the production of fewer suicide haplotypes. Therefore, selfing actually promotes the spread of SDs in this scenario.

Clearly, many factors, including selfing rate, recombination rate, pollen redundancy level, and initial frequency of the killer haplotype, all contribute to the fate of an SD when invading a population of sensitive haplotypes. High pollen redundancy and initial killer haplotype frequency are positive contributors to the spread of an SD, while recombination is a negative contributor. The selfing rate has a complex interaction with the other factors, and can act as either a positive or a negative factor on the spread of an SD.

### Selfing, Recombination and Spread of SD

**Fig. 2.**
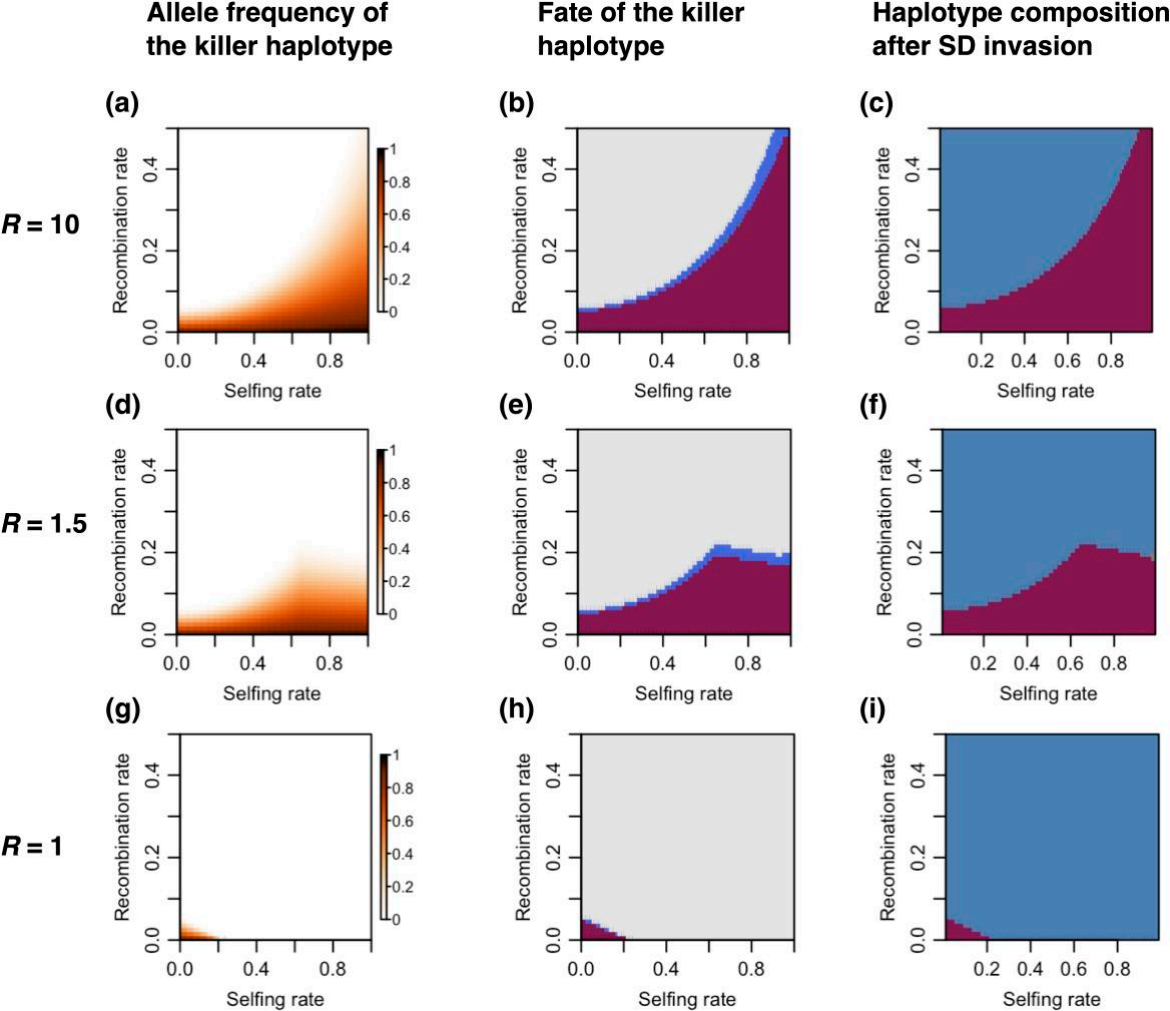
The effect of selfing rate, recombination rate, and redundancy levels on the killer haplotype dynamics. The iterative calculations were performed with the initial frequencies of the sensitive and the killer haplotypes set as 0.9, and 0.1, respectively, and terminated until the proportion of either Ab or aB became <0.001 or until 10,000 generations were completed. The latter is only to put an upper bound on the duration of the iteration, but the former condition is reached nearly always significantly earlier. In the first column of the panel a), d), g), the color shows the allele frequency of the killer haplotype. In the second column b), e), h), the color shows the fate of the killer haplotype at the end of the numerical iterations, with red implying increased in frequency; blue, decrease in frequency; and gray implying that the killer allele was removed from the population. In the third column c), f), i), the color shows the state of the population at the end of the calculations. Red, the killer and the neutral haplotypes coexist; blue, the sensitive and the neutral haplotypes coexist. The first row shows simulation results with pollen redundancy level set to *R* = 10, meaning that the fertility is either weakly or not affected by segregation distortion a) to c). The second row *R* = 1.5, where fertility is partially affected by segregation distortion d) to f). The third row *R* = 1, where fertility is fully affected by segregation distortion g) to i).

The modeling above suggests that the interaction between recombination and selfing rate varies with pollen redundancy levels to determine the evolutionary dynamics of SD invasion. We therefore explored the interaction under different levels of pollen redundancy (Fig. 2). We first fixed pollen redundancy at a high level (*R* = 10) to investigate the intricate interplay between recombination and selfing rate that determines haplotype dynamics by calculating the expected frequency of the killer haplotype under different assumptions regarding recombination and selfing rates (Fig. 2a-c). Under the SD invasion scenario, where a population with the sensitive haplotype is invaded by individuals with the killer haplotype, we first note that a low-recombination rate between the two loci is critical for the killer haplotype to increase in frequency (Fig. 2b). When the recombination rate is greater than 0, the neutral haplotype will emerge and increase in frequency to coexist with other haplotypes (Fig. 1c). And when the recombination rate is lower than a threshold (∼0.05 in this case, Fig. 2b), the killer haplotype frequency will always increase as the fitness increase of the SD allele, owing to the killing effect in the heterozygous state, is larger than the negative fitness effect of forming suicide haplotypes after recombination. A complex interaction between the selfing and recombination rates occurs, with higher selfing rates facilitating the spread of the SD allele even when recombination rates are high (Fig. 2c). This is a result of high-selfing rate inhibiting the formation of the neutral and suicide haplotypes through reducing the effective recombination rate (Nordborg and Donnelly 1997; Conway et al. 1999; Nordborg 2000) between the two loci of the killer haplotype, which partially explains why increased selfing rate is beneficial for the spread of the killer haplotype under the scenario of high pollen redundancy.

### Effect of Gamete Redundancy on the Spread of SDs

In the plant systems considered in this study, reproduction in one flower is associated with one female gamete and thousands of male gametes packed in numerous pollen grains. One pollen is required to fertilize the flower, therefore, there is more pollen than needed for successful fertilization and there is an excess supply of pollen for each flower. The huge redundancy leads to the consequence that elimination of the alternative allele, i.e. abortion of half of the pollen, does not necessarily reduce the fertility of the flower. But it does increase the fertilization chance of the killer haplotype, and increases the absolute number of offspring carrying the killer haplotype. If the pollen number is strictly proportional to the chance of fertilization, then elimination of alternative alleles will not increase the number of offspring carrying the killer haplotype and the killer haplotype will not gain any advantage to spread. Therefore, gamete redundancy is a key for the spread of the killer haplotype. This is akin to the conclusion of Lyttle (1991), which requires segregation distortion to have not just a relative advantage but to be associated with an absolute increase in the number of offsprings carrying the killer haplotype. To illustrate this, we repeat our modeling with the pollen redundancy level set as 1, i.e. the fertility of a flower is proportional to the number of available pollen grains (Fig. 2g-i). In this case, in the majority of the parameter space of recombination and selfing rates, the killer allele will be eliminated while the sensitive haplotype goes to fixation, largely because of the lower fitness associated with the lower fertility of heterozygous individuals carrying both the killer and the sensitive alleles (Fig. 2g-i). Only in the small part of the parameter space where both selfing and recombination rates are low (Fig. 2g-i) and the fertility of the heterozygote is rescued by pollen from the pollen pool will the killer haplotype increase in frequency.

These results provide further insights into the mechanism of how selfing could facilitate the spread of SD under the scenario of high pollen redundancy: due to the unique feature of a perfect flower, when the flower is selfing, there is typically an excess supply of male gametes. The reduction of male gametes because of segregation distortion does not affect the fertility of the individual flower, and therefore, the killer haplotype gains a large, absolute advantage by eliminating pollen with the sensitive haplotype. In an out-crossing scenario, the killer haplotype will join the pollen pool to compete with other haplotypes, the chance to fertilize other flowers is proportional to the number of pollens, in which SDs could only gain marginal advantage by changing the haplotype composition of the pollen pool—an effect providing less of an advantage as the scenario in the selfing mode. For example, in the case of complete selfing, and with complete killing of a sensitive haplotype, the SD allele will double its expected number of offsprings from male gametes in the next generation if in the heterozygous state with a sensitive haplotype. However, if it is competing in a pollen pool with other plants producing *N* pollens and is itself producing *n* pollens, with gamete killing, the proportion of pollens from the SD allele in the heterozygous state with a sensitive haplotype is (*n*/2)/(*n*/2 + *N*) compared with a proportion of (*n*/2)/(*n* + *N*) without segregation distortion; a very small fitness advantage if *n* << *N*. Through this “pollen sequestration” effect, selfing also promotes the spread of the killer haplotype under the scenario of high pollen redundancy.

Moreover, there is an interesting interaction between the pollen redundancy level and selfing rate in determining the spread of SDs: under high pollen redundancy (*R* = 10, Fig. 2a-c), a high-selfing rate is always beneficial for the spread of SDs; while in the absence of pollen redundancy (*R* = 1, Fig. 2g-i), SDs can only spread when the selfing rate is low; at an intermediate redundancy level (*R* = 1.5, Fig. 2d-f), selfing can either promote or hinder the spread of SDs depending on the recombination rate. These intriguing dynamics occur due to a subtle balance of three components: (i) the recombination suppression effect by selfing, which is independent of pollen redundancy level; (ii) the “pollen sequestration” effect, which depends on pollen redundancy level and diminishes when there is no pollen redundancy; and (iii) the fertility reduction effect, which comes into play at low pollen redundancy. At a low selfing rate, the pollen redundancy is dictated by the number of pollen grains from the general pool, i.e. the *M*_pool_ in Eqn. 1, which is not affected by segregation distortion in our model (supplementary table S3, Supplementary Material online), and this diminishes the fertility reduction effect. In summary, at high redundancy levels (*R* = 10, Fig. 2a-c), selfing promotes the spread of SD through recombination suppression and “pollen sequestration” effects; while at a low redundancy level (*R* = 1.5, Fig. 2d-f; *R* = 1, Fig. 2g-i), the “pollen sequestration” effect diminishes, and high-selfing suppresses recombination but increases the fertility reduction effect, which could have both positive and negative effects on the spread of SD.

### Initial Frequency and Spread of SD

In the models above, we noticed that the fate of the killer haplotype could be affected by its initial frequency during invasion (Fig. 1c, supplementary fig. S3, Supplementary Material online). To fully explore this, we modeled the killer haplotype invasion with high pollen redundancy and low initial frequency (*f* = 0.001, supplementary fig. S5, Supplementary Material online). In this case, the spread of SD also relies on a low-recombination rate between the two loci and a high-selfing rate, similar to the case with high initial frequency (*f* = 0.1, Fig. 2a-c), but there is a much smaller part of the parameter space in which the killer haplotype can increase in frequency. For example, when the recombination rate between the two loci is ≥0.01, the killer haplotype will not be able to increase in frequency in the population unless the selfing rate is higher than ∼0.19 for our parameter settings (supplementary fig. S5a-c, Supplementary Material online); while with an initial frequency of 0.1, the SD can spread as long as the selfing rate is >0 (Fig. 2a-c). When starting with an even higher frequency (*f* = 0.5, supplementary fig. S5d-f, Supplementary Material online), SD will be able to increase in frequency in the majority of the parameter space (supplementary fig. S5e, Supplementary Material online) and can always eliminate the sensitive haplotype irrespective of recombination and selfing rates (supplementary fig. S5f, Supplementary Material online). Moreover, with a higher starting frequency, the SD also spreads much faster (supplementary fig. S9a-c, Supplementary Material online).

Whether an SD will increase in frequency from generation to generation, under full pollen redundancy, is determined by a balance between the positive effects of killing competing sensitive haplotypes and the negative effects of suicide haplotype formation. The destruction of competing haplotypes by the killer only results in an allele frequency increase when the relative loss of the sensitive haplotype is greater than that of the killer haplotype, which is frequency-dependent. The per generation change in allele frequency of an SD is positively correlated with its frequency, with high frequencies being beneficial for SD invasion.

**Fig. 3.**
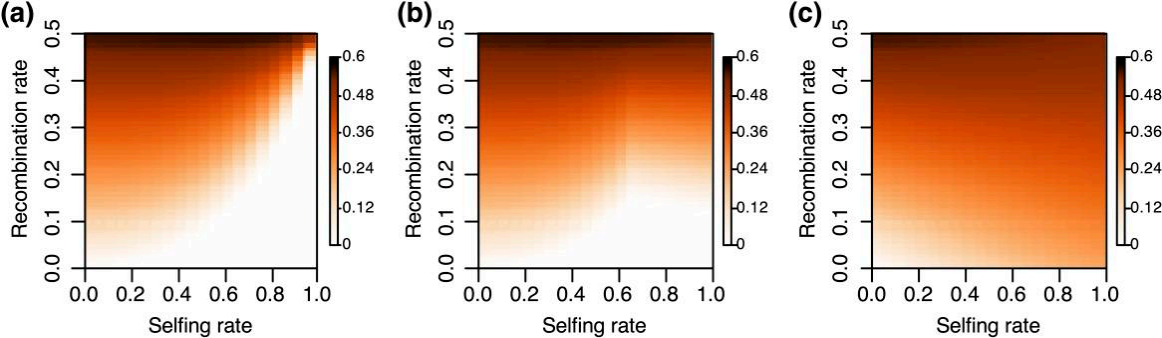
Minimum initial frequency needed for the killer haplotype to spread under different pollen redundancy levels. The pollen redundancy levels are set as a) 10, b) 1.5, and c) 1, respectively. The color corresponds to the value of the initial frequency. The killer was determined to be able to spread if the allele frequency after 100 generations was greater than the initial frequency.

We explored how the initial frequency interacts with recombination rate, selfing rate, and pollen redundancy level to determine the spread of the SD by calculating the minimum allele frequency needed for an SD to spread (Fig. 3). We observe a clear trend in which, under unfavorable conditions, such as low selfing rate, high recombination rate, or low pollen redundancy, higher minimum allele frequencies are required to enable an SD to spread (Fig. 3a-c). With high pollen redundancy (*R* = 10) and a fixed selfing rate, there is a linear-like relationship between minimum allele frequency and recombination rate (supplementary fig. S6a, Supplementary Material online); with a fixed recombination rate, the minimum allele frequency stays relatively constant at low selfing rates but exhibits a steep decline at high-selfing rates (supplementary fig. S6b, Supplementary Material online).

The observation that the spread of an SD is dependent on the initial frequency has important implications for the origin of SDs. Our calculations also show that for most of the parameter space, the initial frequency required for the SD to spread is nonnegligible (Fig. 3), suggesting SD is unlikely to emerge as a new mutation in the population, as new mutations are associated with very low initial frequencies. To further demonstrate this, we ran simulations mimicking SDs arising as new mutations (supplementary fig. S7 to S8, Supplementary Material online). In the populations consisting of neutral and sensitive haplotypes, the killer haplotype can be formed through the gaining of a mutation on the neutral haplotype. When the killer haplotype emerges in the population, it spreads by eliminating sensitive haplotypes. The conditions favorable for the killer to spread are similar to that when the killer allele invades a population with only sensitive haplotypes (supplementary fig. S8, Supplementary Material online and Fig. 2a-c). The killer haplotype is only likely to arise by mutation if the allele frequency of the neutral haplotype is high, but a high neutral haplotype frequency slows down the spread of the killer haplotype. It will take longer for a killer haplotype to reach equilibrium even if it can spread (supplementary fig. S9d, Supplementary Material online), which will subject the killer haplotype to more stochasticity in the presence of genetic drift. Moreover, the maximum frequency of the killer haplotype is capped by the initial frequency of the sensitive haplotype (supplementary fig. S8a, Supplementary Material online).

### SDs Frequently Cross Species Boundaries in Rice Genus

**Fig. 4.**
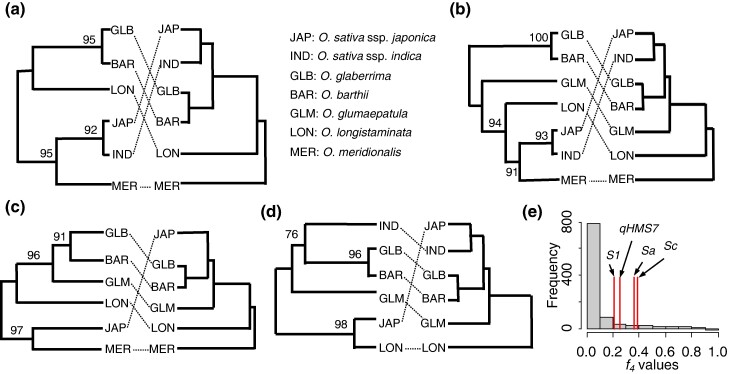
Introgression at the segregation distortion loci in the *Oryza* genus. a) *S1*; b) *qHMS7*; c) *Sc*; d) *Sa*. Left, the gene tree at each locus; right, the species tree. The trees were constructed with ∼1-kb sequence at each segregation distortion loci using maximum-likelihood (see Methods section for details), and the bootstrap values were based on 1,000 replicates. The dash lines connect the same species in different phylogenies. Figure 5b–d have the same abbreviations for different species as shown in Fig. 5a. e) The segregation distortion loci are *f*_4_-value outliers. The graph shows the distribution of *f*_4_ scores of randomly drawn 1-kb regions from the rice genome, and the red vertical lines show the *f*_4_ values of the segregation distortion loci.

Our analyses above show that SDs are unlikely to emerge as new mutations in the population. However, they have a high chance of spreading if they are introduced at moderate or high initial frequencies (Fig. 3), suggesting they are likely to occur during hybridization between different populations, where two populations are dominated by different haplotypes. We investigated all the molecularly characterized SDs associated with pollen abortion in rice (*Oryza sativa*) (Long et al. 2008; Shen et al. 2017; Yu et al. 2018; Xie et al. 2019; Wang et al. 2023a; Wang et al. 2023b; You et al. 2023; Zhou et al. 2023), a highly selfing species, and its closely related wild *Oryza* species. Surprisingly, at all of the four loci where sequences could be retrieved from different *Oryza* species to perform phylogenetic analyses (supplementary Text S1, Supplementary Material online), including *qHMS7* (Yu et al. 2018), *Sa* (Long et al. 2008), *S1* (Xie et al. 2019), and *Sc* (Shen et al. 2017), the local gene trees have topologies that are drastically different from the species tree (Fig. 4), showing strong evidence of introgression across different species. At *S1*, Asian cultivated rice (*O. sativa*) forms a sister clade with the basal *Oryza* species of AA genome, *Oryza meridionalis* (Fig. 4a, bootstrap value = 95). This pattern is unlikely to be caused by incomplete lineage sorting as the chance that one allele was sorted into both the basal *O. meridionalis* and Asian cultivated rice (*O. sativa*) lineages but not other lineages is very low. However, this pattern is consistent with an introgression from *O. meridionalis* into the common ancestor of Asian cultivated rice. Similarly, local phylogenies at *Sc* and *Sa* are consistent with introgressions from *O. meridionalis* into the *O. sativa* and *O. longistaminata* into *O. sativa*, respectively (Fig. 5c-d). And introgression between *O. meridionalis* and *O. sativa* has occurred at the *qHMS7* locus, as these two species form a well-supported clade (bootstrap value = 91) that is markedly incongruent with the species tree (Fig. 5b). These results provide strong evidence for introgressions across different species at all these loci, suggesting these SDs underwent a history of hybridization between different species and crossed species boundaries as a consequence. To evaluate whether the introgression signals at these segregation distortion loci could occur randomly given the background level of introgression in the genome, we obtained the distribution of *f*_4_ values (Lipson 2020) for randomly drawn regions across rice genomes and compared it with those observed at the segregation distortion loci (Methods). All the *f*_4_ values of the four loci are outliers. Specifically, they are ranked in the 12.8% (*S1*), 11.3% (*qHMS7*), 8.5% (*Sa*), and 8.1% (*Sc*) percentile at the genome-wide level (Fisher's combined *P*-value = 0.011), respectively, supporting that introgression is more likely to occur at these segregation distortion loci than at random genomic regions. Therefore, these segregation distortion loci, which were initially identified as hybrid incompatibilities, and were presumed to hinder gene-flow between populations, paradoxically, may in fact be important drivers of introgression as they promote the introgression of genomic regions in linkage with them.

## Discussion

In this study, we have shown that selfing as a form of inbreeding actually can promote the spread of SDs in hermaphroditic plants, contrary to what was previously believed (Burt and Trivers 1998; Fishman and McIntosh 2019). Our model differs from previous ones in an important aspect: we consider a two-loci system underlying an SD, while previous studies modeled an SD as an extra, nonrecombining chromosome (Burt and Trivers 1998), or as a one-locus system (Bull 2016; Bull et al. 2019). Our results are in fact not contradictory to previous ones, but highlight the interesting observation that the effect of inbreeding on the spread of SD may vary depending on the specifics of the genetic architecture. In a two-loci system, genes at the two loci underlying an SD are both indispensable components of SDs found in hermaphroditic plants, which are often described as “toxin–antidote” systems with one gene corresponding to the toxin and the other corresponding to the antidote. Our model is relevant in the context of numerous recent studies that characterized genic SDs at the molecular level and showed that they are often caused by multiple genes (Burga et al. 2020; Sweigart et al. 2019). The linkage between haplotypes of these genes is the key for a viable SD. We note that inbreeding or selfing reduces the effective recombination rate (Nordborg and Donnelly 1997; Conway et al. 1999; Nordborg 2000) between the two loci in the population, thereby facilitating the driving of SDs by inhibiting the break-down of the SD haplotype. Moreover, it has long been recognized that SDs tend to locate in recombination suppressed regions. A famous example is the Sd/Rsp system in Drosophila, which consists of two genes located on either side of a centromere (Wu and Hammer 1991). The t-haplotype in mice is a recombination block caused by inversions (Hammer et al. 1989). In fungi, numerous identified SDs are almost always associated with complicated sequence rearrangements including inversions that suppress local recombination (Turner and Perkins 1979; Zanders et al. 2014; Svedberg et al. 2018). In plants, different components of many characterized SDs are found to be in close physical proximity (Long et al. 2008; Yang et al. 2012; Kubo et al. 2016; Shen et al. 2017; Yu et al. 2018; Xie et al. 2019) or in centromere region (Finseth et al. 2021; Fishman and Saunders 2008), rendering them seemingly “one-locus’. Inbreeding is another alternative mechanism that will reduce recombination between different components of an SD, thereby facilitating its spread.

Our analyses also highlight gamete redundancy as another crucial factor for the spread of SDs. More generally, the spread of SDs depends on gamete redundancy or sibling redundancy. SDs are often associated with the destruction of gametes of an individual or killing of siblings carrying alternative alleles, which is detrimental to the host. Intuitively, they should be purged rapidly from the population. However, with gamete or sibling redundancy, the detrimental effect of SDs can largely be buffered at the individual or population level, and selection will fail to remove these “selfish” loci. Consequently, it is not surprising that the majority of identified SDs in plants are pollen killers, as they have minimal impact on the fertility of a flower due to gamete redundancy. Similarly, in animals, segregation distortion is also more likely to occur in male gametes (Lyttle 1991), as the large number of male gametes leads to redundancy, which mitigates the detrimental effect of SDs on fertility. Similarly, with strong local sibling competition and redundancy, SDs that reduce an individuals’ offspring number do not necessarily reduce their fitness. In fungi, the retaining of SDs that eliminate haploid offspring in the population can be attributable to strong local sibling competition (Nauta and Hoekstra 1993). In plants, one individual often produces a large number of offsprings that are distributed in a relatively small space, leading to strong sibling competition or redundancy. This may explain why SDs acting as female gamete killers are also frequently detected in plants, e.g. the *S5* (Yang et al. 2012) and *HSA1* (Kubo et al. 2016) loci that abort female gametes in rice.

The spreading of SDs is frequency-dependent and SDs are more likely to spread with high initial frequency. Therefore, SDs are more likely to spread in scenarios where populations with different haplotypes hybridize, as opposed to emerging as new mutations. When SDs are involved in hybridization involving many matings, they will begin at intermediate allele frequencies, while SDs arising as new mutations will initially have extremely low allele frequencies. Our empirical analysis in rice at previously characterized SD loci confirmed that all SDs had histories of population hybridization and crossed species boundaries as a result, although further population genetic data are needed to determine whether the direction of these introgressions is consistent with the hypothesized scenario in which the killer haplotype invaded the population with sensitive haplotypes. Similar observations have also been made in other systems (Meiklejohn et al. 2018; Svedberg et al. 2021), suggesting the hybridization history may be a common feature associated with these genic SDs. These results also have important implications for the origin of these seemingly “selfish” genetic elements, supporting that they may originate simply as Bateson–Dobzhansky–Muller incompatibilities (BDMIs) in a nonselfish manner in isolated populations, as also suggested by Sweigart et al. (2019). The selfish behavior (gamete killing) may be a manifestation of hybrid incompatibility when previously isolated populations meet.

In this work, we have not investigated how factors such as recombination, selfing rates, and pollen redundancy might be evolvable characters. Rather, we have focused on the fate of SDs in a toxin–antidote system with fixed values of these parameters. However, we note that much previous work has investigated the evolution of these characters in the context of selfing. For example, Charnov investigates male/female allocation as a function of selfing rate (Charnov 1987), and such trade-offs could also be investigated in the current system as a modifier of pollen redundancy. Previous studies have also argued that selfish elements drive the evolution of low-recombination rates (Thomson and Feldman 1974; Charlesworth and Hartl 1978; Burt and Trivers 2009; Charlesworth and Charlesworth 2010; Haig 2010). While this might be possible, a perhaps more likely explanation for the observation that selfish genetic elements tend to appear in low-recombination regions is an ascertainment bias caused by only observing the SDs that arise in low-recombination regions. A large number of hybrid incompatibilities may be present when divergent populations hybridize, and only the ones with loci that are closely linked are able to spread and be maintained in the population.

Our study highlights the possibility that segregation distortion might be driving introgression, particularly in selfing plants, and can arise as simple BDMIs. While BDMIs generally are considered barriers to gene-flow, they may in fact be drivers of gene-flow when causing haplotype-specific pollen killing.

## Methods

### Plant Model

#### Flower Structure and Gamete Numbers

The vast majority of flowering plants are hermaphroditic, which means they have both sexes in one flower. Here we consider a typical hermaphroditic plant system with perfect flowers. Each flower has a female reproductive organ, the pistil, that contains one or a few female gametes that ultimately form the embryo, and a male reproductive organ, the stamen, that contains a large number of male gametes. For example, in rice, each flower has one pistil that contains one female gamete that can develop into an embryo, and six stamens each containing thousands of pollens (Nakamura et al. 2000), where each pollen contains one male gamete. While such a morphology, in which each flower has just a single female, is rare in most plants, the feature of the system that matters for the modeling predictions is the extraordinarily high redundancy of male gametes.

#### Mating System and Inbreeding Coefficient

Genetically, inbreeding is the outcome of zygote formation combining two sets of closely related genomes. Biologically, inbreeding has different causes depending on mating systems. In strictly out-crossing species, matings between closely related individuals lead to increased levels of inbreeding in the population. In species that are able to self-fertilize, zygote formation through combination of gametes from the same individual, i.e. selfing, represents one form of inbreeding. In addition to selfing, the cross-fertilization between different individuals that are closely related also contributes to inbreeding in hermaphroditic species. However, we use the selfing rate as a proxy for the probability that two alleles in an individual are identical by descent (the inbreeding coefficient), as it is the major contributor of inbreeding in a hermaphroditic plant system where zygotes can be formed through either selfing or cross-pollination.

When a plant selfs, it receives pollen from the same flower or other flowers of the same individual; when it cross pollinates, it receives pollen from other plants. Here we define the selfing rate for each flower as the probability that this flower receives pollen from the same individual, which could be from the same flower or a different flower of the same individual (including both autogamous and geitonogamous self-pollination), a setup that was also adopted in models studying the evolution of plant mating systems (Gregorius et al. 1987; Holsinger 1991). Let the total number of pollen grains that can access a flower produced by the plant of that flower be *M*_self_. We refer to the pool of pollen that can potentially fertilize each flower through cross-pollination as the “pollen pool”. Let the number of pollens that can access the flower from the pollen pool be *M*_pool_. Assuming equal pollination probability of all pollens that can access the flower, the selfing rate, *s*, is thus defined as follows:


(1) $$s\;=\;\frac{M_{\mathrm{self}}}{M_{\mathrm{self}}+M_{\mathrm{pool}}}.$$

### SD Model

Here we consider a two-loci (loci 1 and 2) system underlying the segregation distortion that takes place during male gametogenesis. The recombination rate between the two loci is denoted by *r* (0 ≤ *r* ≤ 0.5). There are two alleles at each locus A/a for locus 1 and B/b for locus 2, respectively. We assume male gametes bearing the “a” allele will be killed, with a probability of *k* (0 ≤ *k* ≤ 1), in the presence of the “b” allele in the individual that produced this pollen. We did not explore the impact of this parameter on the dynamics of segregation distortion but fixed *k* as 1 throughout our simulations. Note that incompatibility could occur between sporophytes and gametophytes, and it is not required that alleles “a” and “b” be on the same haplotype. In total, there are four possible haplotypes denoted as follows:

- AB - “neutral”, this haplotype cannot be killed and does not kill another haplotype,
- Ab - “killer”, it kills the other haplotype if that haplotype carries “a” but cannot itself be killed,
- aB - “sensitive”, it can be killed if the other haplotype carries “b” but does not kill any haplotype,
- ab - “suicide”, this haplotype can kill itself and can also kill the other haplotypes.

Note that the suicidal haplotype can be present in a population only if *k* does not equal 1. This model simulates the SDs of the toxin–antidote system (Burga et al. 2020) often constituting two or more closely linked genes, which is the form of genic SDs frequently found in plants (Long et al. 2008; Yang et al. 2012; Shen et al. 2017; Yu et al. 2018; Xie et al. 2019). The toxin and antidote correspond to “b” and “A” alleles at the loci in our model, respectively. With these four haplotypes, there are 10 different diploid combinations of haplotypes, which are given in the ***H*** vector as follows:


$$\begin{array}{rl} H= & (AB|AB,AB|Ab,AB|aB,AB|ab,Ab|Ab,Ab|aB,\\ & \;Ab|ab,aB|aB,aB|ab,ab|ab) \end{array}.$$

The combination “AB|ab” and “Ab|aB” have the same genotypes in the two loci, but have different phases, and we distinguish between them as they generate different distributions of haplotypes in the pollen. We denote the *i*th component of ***H*** by ***H****_i_*, and the first haplotype of ***H****_i_* by ***H****_i_*^1^ (resp. the second haplotype of ***H****_i_* by ***H****_i_*^2^).

#### Gamete Generating Matrices

As SD only affects male gametes, the male and female gamete generating matrices are not equal. With the segregation distortion model defined above, we can write down both the male ***G*^m^** and female ***G*^f^** gamete generating matrices. Each row of the matrix corresponds to one of the 10 possible genotypes and each column to one of the four possible haplotypes. The entry *G*^m^*_i,j_* (resp. *G*^f^*_i,j_*) corresponds to the probability of a male (resp. female) gamete from an individual of genotype *i* to carry the haplotype *j* and not to be killed. The male and female gamete generating matrices are given in supplementary table S1 and S2, Supplementary Material online. Without recombination or segregation distortion, for a genotype x|y, the corresponding row in the matrix would have all 0 s, except for the columns corresponding to haplotypes x and y, which would both have the value 0.5, according to Mendel's first law. In the presence of recombination at rate *r* and for a genotype, say AB|ab as an example, the probability of a gamete carrying AB or ab is 0.5(1–*r*), and the probability of a gamete carrying Ab or aB is 0.5*r*.

Finally, taking segregation distortion (as described above) into account, for genotype AB|ab, the relative contribution of a male gamete carrying ab (resp. aB) is 0.5(1–*r*)(1–*k*) [resp. 0.5*r*(1–*k*)] as male gametes carrying allele “a” are killed with rate *k* when the flower carries at least one copy of the allele “b” as well. Similar logic applies to the other entries of ***G*^m^** and ***G*^f^**.

Consider the vector ***θ*** of the genotype frequencies in the current generation. The haplotype frequencies of the pollen pool in this generation are then given by the vector ***f,*** which is the normalized product of the male gamete generating matrix ***G*^m^** and the vector ***θ***:


(2) $$f=\frac{G^{m}\theta }{\left||G^{m}\theta |\right|}.$$

#### Fertility and Pollen Number

For each female gamete, one pollen is adequate to fertilize it to form an embryo of the next generation. Therefore, as many more pollens than female gametes are being produced, there is typically substantial redundancy of male gametes. We define for genotype *i*, the fertility *Φ_i_*, as the probability of successfully forming an embryo that contributes to the next generation given available pollen numbers and we consider it only to be a function of the pollen number. We introduce a parameter, *R* ≥ 1, representing the pollen redundancy level, which can be defined as the number of pollen grains delivered to a flower during fertilization divided by the number of pollen grains needed to fertilize all ovules of the flower. The value of *R* can be set arbitrarily to model pollen supplies in different mating systems. For a flower of genotype *i*, the pollen number *N_i_* represents the amount of pollen available for the flower. It is given by adding the external pollen pool *M*_pool_, which is the same for every flower, to the pollen produced by the flower and not killed by segregation distortion. Recall that the amount of pollen produced by the flower depends on its genotype and it is proportional to the sum of the corresponding row in the male gamete generating matrix ***G^m^***. For each genotype, the total pollen number available for each flower is given by supplementary table S3, Supplementary Material online.

For a normal flower, not affected by segregation distortion, the total number of pollen grains available for this flower is *N_i_,* which is the sum *of M*_pool_ and *M*_self_. When the pollen number is above (*M*_pool_ + *M*_self_)/*R*, we define the fertility to be 1. When the pollen number is lower than (*M*_pool_ + *M*_self_)/*R*, we consider a simple linear relationship between fertility and pollen number. The relationship between fertility *Φ_i_* and pollen number, *N_i_*, can then be written as


(3) $$\phi_{i}=\left\{ \begin{array}{c} \frac{N_{i}\cdot R}{M_{\mathrm{pool}}+M_{\mathrm{self}}},\mathrm{if}\;0\;<\;N_{i}\leq \frac{M_{\mathrm{pool}}+M_{\mathrm{self}}}{R}\;\\ 1,\;\;\;\;\;\mathrm{if}\;N_{i}>\frac{M_{\mathrm{pool}}+M_{\mathrm{self}}}{R}\; \end{array}\right.\;\;.$$

The assumed relationship between fertility and number of pollens is a biologically reasonable and mathematically simple approximation. There could be other ways to define the function (for example as a logistic function rather than a linear function), but it achieves the major goal of capturing the critical feature of high pollen redundancy and increased fertility as the number of the pollen grains increases.

#### Genotype Transition Matrix

To trace the dynamics of these haplotypes in time, we must obtain a genotype transition matrix (***G*** = {*g_ij_*}) defining the probability distribution of offspring genotypes in the next generation for each flower genotype. Multiplying this matrix with the vector containing the frequency of each genotype in the current generation will give us the frequency of each genotype in the next generation.

We first define the probability *p_ij_* that the offspring of a flower of genotype *i* is of genotype *j*, assuming that this flower has been fertilized, as follows:


(4) $$\begin{array}{rl} p_{ij}\;= & P(\mathrm{offspring}\;\mathrm{of}\;\mathrm{genotype}\;j\\ & |\mathrm{flower}\;\mathrm{of}\;\mathrm{genotype}\;i\;\mathrm{is}\;\mathrm{fertilized}). \end{array}$$

The probability *p_ij_* is the probability of a flower of genotype *i* producing a female gamete of haplotype ***H****_j_*^1^ multiplied by the probability of receiving pollen of haplotype ***H****_j_*^2^, plus the probability of the flower producing a female gamete of haplotype ***H****_j_*^2^ multiplied by the probability of receiving pollen of haplotype ***H****_j_*^1^. The probability of receiving a pollen of a certain haplotype is the probability of receiving it from itself (self-pollination) added to the probability of receiving it from the other flowers (cross-pollination). This gives


(5) $$\begin{array}{r} p_{ij}=\left\{ \begin{array}{c} G_{i,H_{j}^{1}}^{f}\cdot \frac{M_{\mathrm{self}}\cdot G_{i,H_{j}^{2}}^{m}+M_{\mathrm{pool}}\;f_{H_{j}^{2}}}{N_{i}},\mathrm{if}\;H_{j}^{1}=H_{j}^{2},\mathrm{else}\;\\ G_{i,H_{j}^{1}}^{f}\cdot \frac{M_{\mathrm{self}}\cdot G_{i,H_{j}^{2}}^{m}+M_{\mathrm{pool}}\;f_{H_{j}^{2}}}{N_{i}}\\ \;\;+G_{i,H_{j}^{2}}^{f}\cdot \frac{M_{\mathrm{self}}\cdot G_{i,H_{j}^{1}}^{m}+M_{\mathrm{pool}}\;f_{H_{j}^{1}}}{N_{i}}\; \end{array}\right.\;\;, \end{array}$$

With $f_{H_{j}^{1}}$ (resp $f_{H_{j}^{2}}$), the frequency of haplotype $H_{j}^{1}\;$(resp. $H_{j}^{2})$ in the pollen pool, given by the vector ***f*** defined at Equation (2). The presence of *N_i_* is such that the probabilities sum to one.

We then have


(6) $$g_{ij}=\phi_{i}\cdot p_{ij}.$$

The full genotype generating matrix ***G*** can be found in supplementary table S4, Supplementary Material online.

### Numerical Calculations

The initial population genotype frequencies were set to mimic the invasion of an SD, i.e. the frequencies of “Ab|Ab” (killer haplotypes) and “aB|aB” (sensitive haplotypes) were set at nonzero values and the rest of the haplotype combinations were initially set to be 0. The killing efficiency parameter, *k*, is set as 1, and different values of *r*, *s,* and *R* are used to model different scenarios. The value of *M*_pool_  *+ M*_self_ can be set arbitrarily as only the ratio (determined by *s*) between *M*_pool_ and *M*_self_ matters.

We then calculate the expected haplotype frequencies by iterating Equation (6) forward in time. As it is difficult to obtain analytical solutions for this system, we trace the dynamics forward in time to investigate the equilibrium conditions. When either the frequency of the sensitive or the killer haplotype drops to 1% of the initial frequency (approximating loss), or the system has been iterated 10,000 generations, we assume equilibrium has been reached. We then record the state of the system in this pseudo-equilibrium according to whether the killer haplotype is lost or fixed (using the 1% of initial frequency criterion).

Note that the killer and sensitive haplotypes cannot coexist in equilibrium, because when a gamete of type Ab fuses with a gamete of type aB, then the allele “a” decreases in proportion compared with “A”. This decrease cannot be compensated by other matings, as no combination of haplotype favors allele “a” compared with “A”. Therefore, there are five possible equilibrium states: the fixation of one haplotype (killer, sensitive, or neutral), coexistence of the neutral and sensitive haplotypes, or coexistence of the neutral and the killer haplotypes.

### Synteny Analysis at Segregation Distortion Loci

The orthologous regions of the segregation loci from each genome assembly (supplementary table S5, Supplementary Material online) were identified using Blat (version 35) (James Kent 2002). And dot-plots were plotted at the syntenic region using R.

### Phylogenetic Analysis

Before tree construction, the sequence alignment was performed using MAFFT (version v7.508) (Katoh and Standley 2013). The tree was constructed using a maximum-likelihood method implemented in IQ-TREE (version 2.0.3) (Minh et al. 2020) with parameters “–alrt 1,000 -m TEST”, and the bootstrap values were based on 1,000 replicates. At the *qHMS7* locus, the Nipponbare genome coordinates for the ∼1-kb syntenic region across all species used for tree construction are Chr7:26989559.26990682; the coordinates at *S1* locus are Chr6:2192850.2193862; Chr1:22377078.22378081 at *Sa* locus; and Chr3:7792688.7793698 at *Sc* locus.

### Genome Alignment

To generate genome alignment at base pair resolution, we first identify syntenic regions using SyRI (Goel et al. 2019) between assemblies based on genome alignments generated using Minimap2 (Li, 2018). Sequences in syntenic regions from different assemblies were further aligned using Blat (version 35) (James Kent 2002).

### Distribution of *f*_4_ Values

The *f*_4_ values (Patterson et al. 2012) were calculated in the form of *f*_4_ (A, B; C, D) where A, B, C, and D refer to four populations. The *f*_4_-statistic has an expected value of 0 when the four populations are related by the unrooted phylogeny of [(A, B), (C, D)] (Lipson 2020). To get an indicator of introgression for a region, we calculated *f*_4_ values for all possible combinations of quadruplets based on the species tree of AA genome *Oryza* species and took the maximum *f*_4_ values as the introgression indicator for the region. The phylogeny of AA genome Oryza species is (*O. meridionalis*, *O. longistaminata*, *O. glumaepatula*, *O. barthii*, *O. glaberrima*, *O. sativa* ssp. *japonica*, *O. sativa* ssp. *indica*) (Ge et al. 1999).

## Supplementary Material

msae132_Supplementary_Data

## Data Availability

No datasets were generated in the current study.

## Code Availability

The description of the models is provided in the Methods section, and the R code used in the simulation of models is available on Github (https://github.com/hrwang/SegregationDistorterModeling).
