# Supplementary material for: Selfing Promotes Spread and Introgression of Segregation Distorters in Hermaphroditic Plants
Source: Mol Biol Evol. 2024 Jun 27;41(7):msae132. doi: 10.1093/molbev/msae132 (PMC11226791; doi:10.1093/molbev/msae132)
Supplement: msae132_Supplementary_Data [file msae132_supplementary_data.docx]

## Supplementary Text

### Supplementary Text 1. Segregation distortion loci analyses in rice (Oryza sativa L.)

There are six SDs associated with pollen killing that have been molecularly characterized, i.e., *Sa* [(Long et al., 2008)](https://paperpile.com/c/afK3iD/wnyxj), *qHMS7* [(Yu et al., 2018)](https://paperpile.com/c/afK3iD/PnVqZ), *S1* [(Xie et al., 2017; Koide et al., 2018; Xie et al., 2019)](https://paperpile.com/c/afK3iD/oxvgK+npzN7+REBDJ), *Sc* [(Shen et al., 2017)](https://paperpile.com/c/afK3iD/yMqTu), *Se* [(Wang et al., 2023b, 2023a; Zhou et al., 2023)](https://paperpile.com/c/afK3iD/Q49Dj+s8piA+qnUwN) and *qHMS1* [(You et al., 2023)](https://paperpile.com/c/afK3iD/CBTZ). The *Se* locus was only found to be present in Asian, African rice and *O. meridionalis* [(Wang et al., 2023b)](https://paperpile.com/c/afK3iD/Q49Dj), and the *qHMS1* sequences were retrievable only from the genome assemblies of *O. longistaminata*, Asian and African rice (Fig. S10), which do not provide adequate phylogenetic signal to determine the status of introgression and thus not included for further phylogenetic analysis. The segregation distortion loci are often associated with complicated sequence rearrangements. To study the sequence variations, we leveraged reference genome or high-quality genome assemblies based on PacBio long-read sequencing technology (Table S5) to retrieve genomic sequences at these loci.

####

#### *Sa* locus

The *Sa* locus was mapped as a locus underlying the hybrid incompatibility between *O. sativa* ssp. *japonica* and *O. sativa* ssp. *indica*. The F1 individual that is heterozygous at this locus shows male sterility as the pollen with the japonica allele was aborted. Genetic mapping identifies two adjacent genes, *SaF* and *SaM*, at this locus and they interact to contribute to the phenotype.

To study the evolutionary history of the locus, we performed synteny analysis in Asian cultivated rice and its closely related AA genome *Oryza* species. Using a high-quality genome assembly panel based on PacBio SMRT read sequencing technology (Table S5), the genomic region orthologous to the ~9-kb genomic region spanning SaF and SaM from Nipponbare, a variety of *O. sativa* ssp. *japonica*, were successfully identified from all species except *O. meridionalis* (Fig. S11). To check whether the absence in *O. meridionalis* is caused by error during genome assembly, we searched the homologous sequence in two other genome assemblies of *O. meridionalis* and confirmed that *Sa* locus is missing in the *O. meridionalis* genome. Extensive structural variations are found within the genome panel, and the most notable ones include large insertions in *O. barthii*, *O. glaberrima* and *O. sativa* ssp. *indica*. Phylogenetic analysis at the locus (Fig. 4) shows that the local phylogeny is inconsistent with the species tree, as the japonica group of *O. sativa* clusters with the *O. longistaminata* which is basal in the species tree. This pattern is consistent with a single introgression event from *O. longistaminata* into *O. sativa* ssp. *japonica*.

#### *qHMS7* locus

The *qHMS7* is a hybrid incompatibility locus in the cross between *O. sativa* and *O. meridionalis*. The F1 individual that is heterozygous at this locus shows male sterility as the pollen with the *O. meridionalis* allele was aborted. Genetic mapping identifies two adjacent genes, *ORF2* encoding the ‘toxin’ and *ORF3* encoding the ‘antidote’, at this locus. The *ORF3* is missing from the *O. meridionalis* haplotype.

Synteny analysis of the *qHMS7* locus revealed extensive structural variation in this region and confirmed the absence of *ORF3* in the *O. meridionalis* genome (Fig. S12). In fact, *ORF3* is specific to *O. sativa* lineage. The other notable variation is a large duplication of sequence spanning the *ORF2* and *ORF3* in Minghui63 genome (*O. sativa* ssp. indica), the high sequence identity between the two copies of sequence suggest that the duplication is a recent event in the Minghui63 lineage. With the assistance of the synteny analysis, we constructed a local phylogeny with ~1-kb orthologous sequence across all species (Fig. 4). The phylogeny is consistent with an introgression from *O. meridionalis* into the common ancestor of *O. sativa*.

#### *S1* locus

The *S1* is a hybrid incompatibility locus mapped in the cross between *O. sativa* and *O. glaberrima*. There are three closely linked genes (*S1A4*, *S1TPR*, and *S1A6*) in the *O. glaberrima* haplotype. The three genes collectively generate the killing signal, while the functional *TPR* gene is protective against the killing. In the heterozygous carrying both *O. sativa* and *O. glaberrima* allele, the pollen with the *O. sativa* allele is aborted as it does not harbor a functional *TPR* gene.

Synteny analysis at the locus shows extensive structural variation in the region (Fig. S13). The orthologous regions cannot be identified in the *O. glumaepatula* genome assembly. The orthologous regions of *S1A4* are missing in *O. sativa*, *O. longistaminata* and *O. meridionalis* genome assemblies, and the orthologous regions of *S1A6* are missing in *O. sativa* and *O. longistaminata*. The *S1TPR* gene region is relatively conserved, with orthologous sequences identified across different species. With the assistance of the synteny analysis, we constructed a local phylogenetic tree using orthologous sequence within *S1TPR* (Fig. 4). The tree shows *O. sativa* and African rice (*O. barthii* and *O. glaberrima*) are clustered with the two basal species, *O. meridionalis* and *O. longistaminata*, respectively. The tree is unlikely to be caused by incomplete lineage sorting, but probably caused by introgression across different rice groups. The most parsimonious scenario is an introgression from the basal species (*O. meridionalis*) into the common ancestor of *O. sativa* groups.

####

#### *Sc* locus

The *Sc* locus was mapped as a hybrid incompatibility locus in the cross between the two subspecies of *O. sativa*, and in individuals that are heterozygous at the locus, pollen with the japonica allele was aborted. The hybrid male sterility was conferred by structural variation, as the indica allele harbors multiple copies of 28-kb tandem duplicates while the japonica allele contains one copy. Each copy of the interval contains a pollen essential gene encoding DUF1618 domain protein.

Synteny analysis of the locus using the genome panel consisting of different *Oryza* species confirmed the presence of ~28-kb tandem duplicates in *O. sativa* ssp. indica (Fig. S14). The duplications are probably specific to the indica lineage as they are not found in other species. Other notable structural variants at the locus include a ~5-kb insertion in *O. longistaminata*, and a ~2-kb tandem duplication within the DUF1618 domain protein coding gene. A phylogenetic tree constructed with fragments in the gene (Fig. 4) shows that *O. sativa* clusters with the basal species, *O. meridionalis*. This result is most consistent with a scenario involving introgression from *O. meridionalis* into *O. sativa* at the locus.

##

## Supplementary Tables

**Table S1**. Male gametes-generating matrix G^m^.


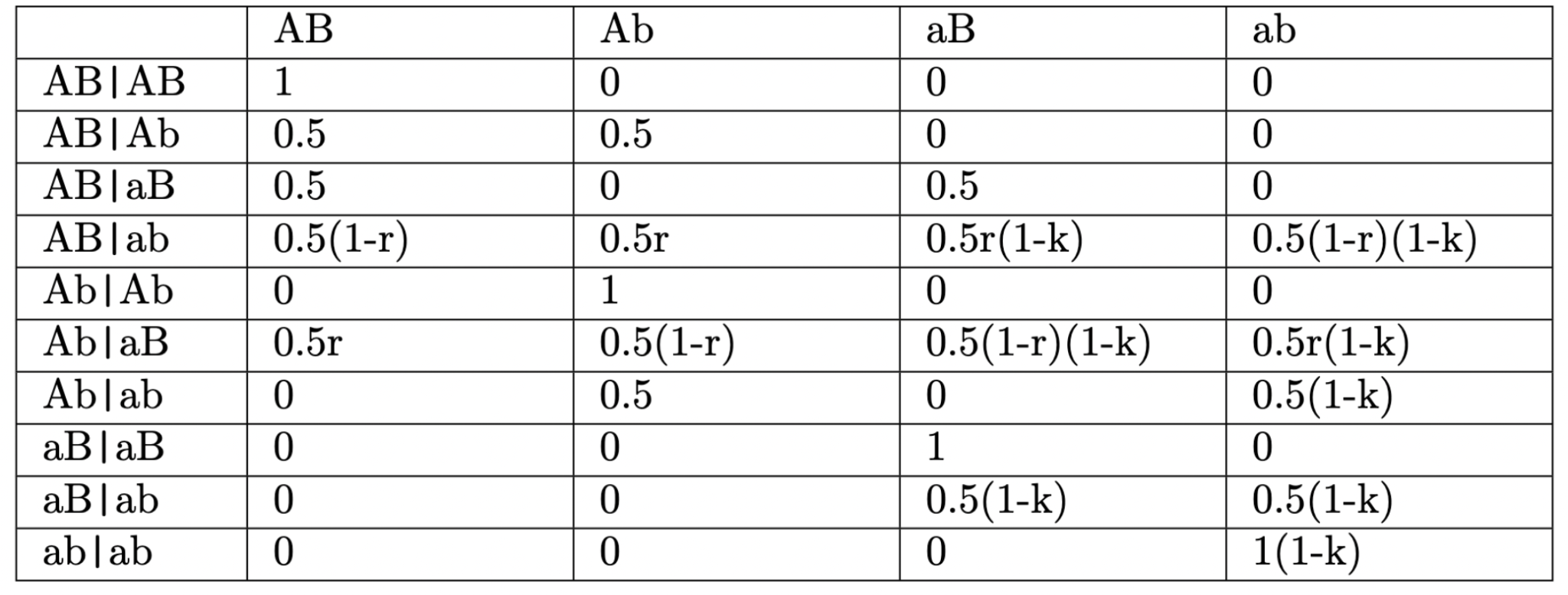


**Table S2**. Female gamete generating matrix G^f^.


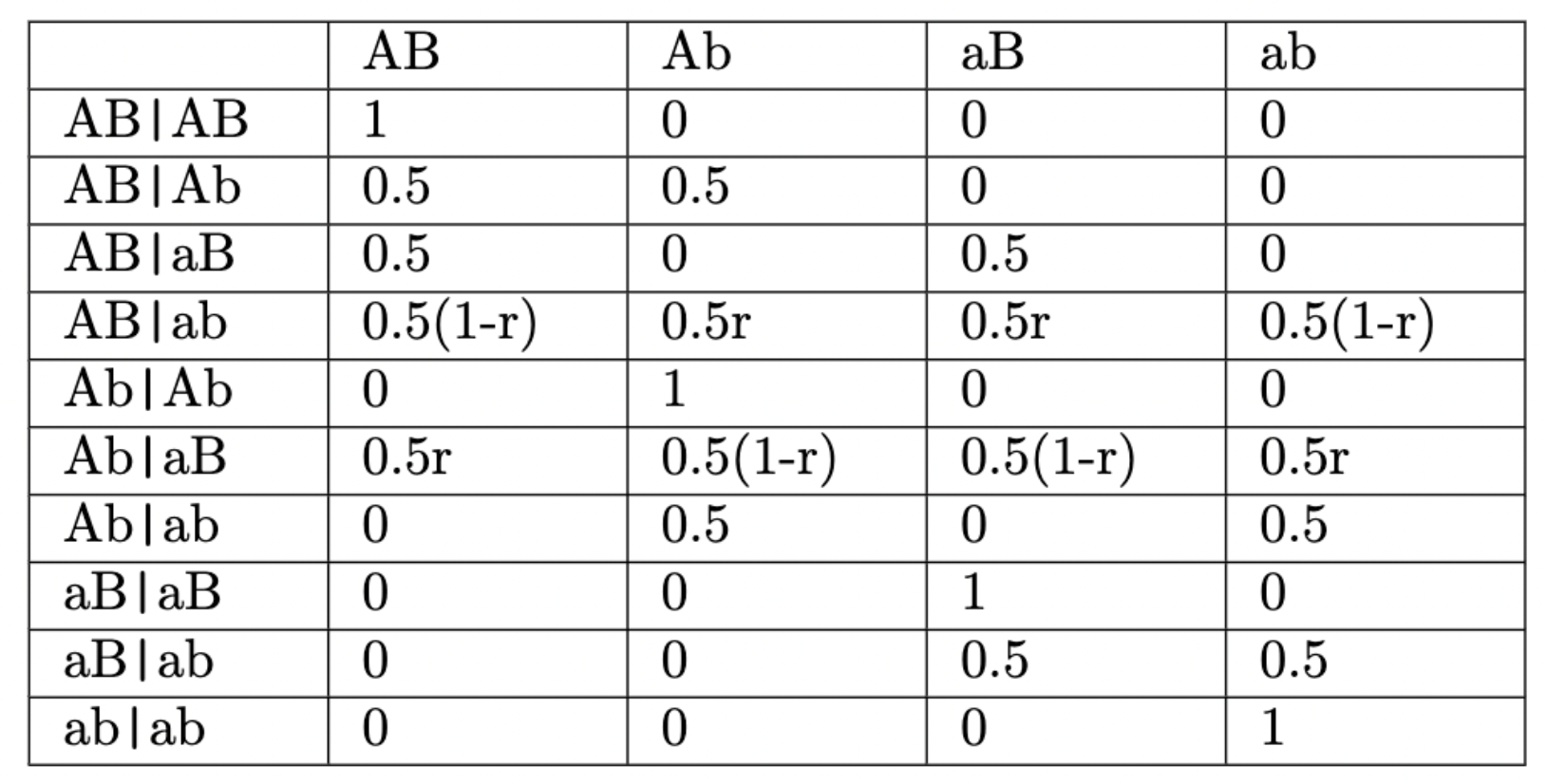


**Table S3**. Number of pollen (*N*) available for each flower haplotype.


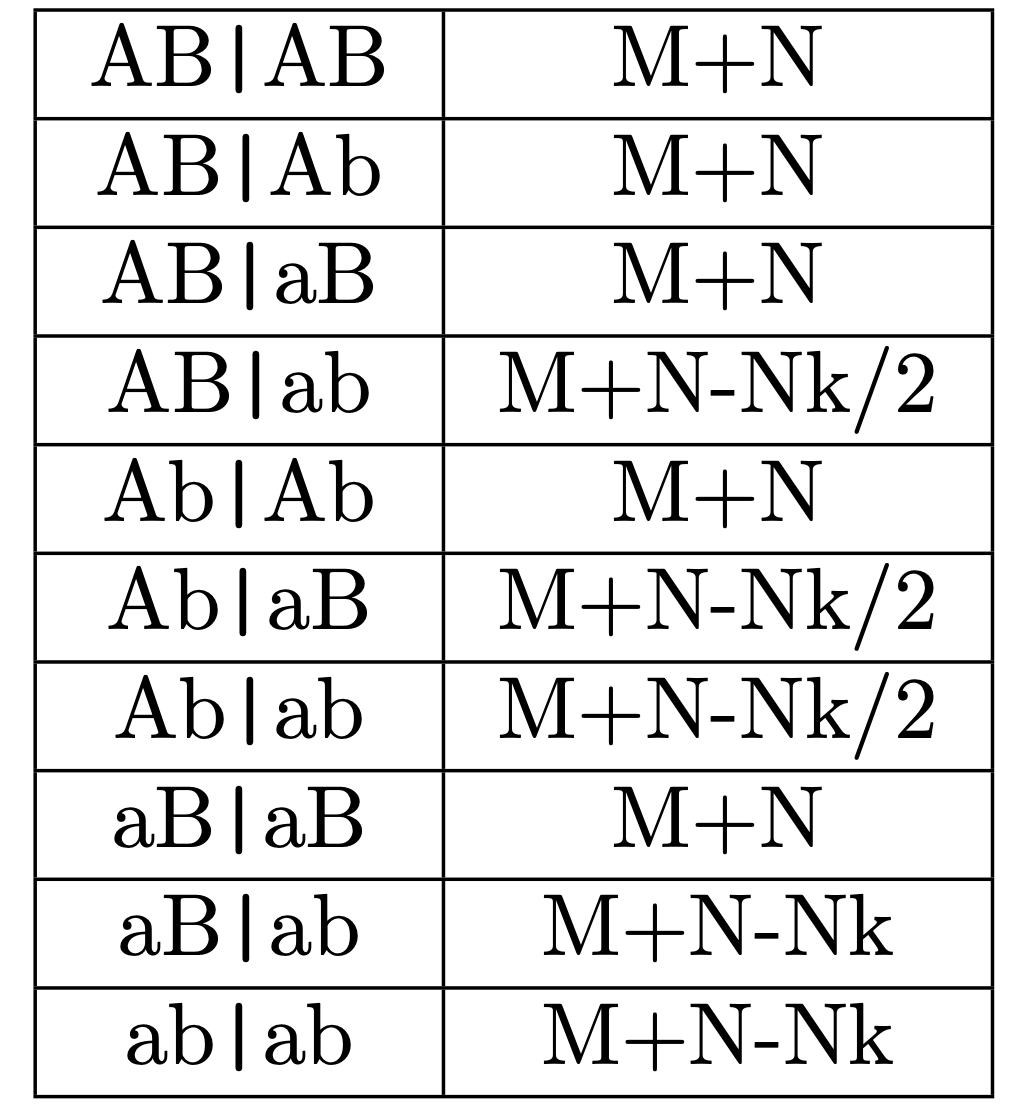


**Table S4**. The genotype generating matrix G. The value of *N_i_* can be found in Table S3. The matrix is split into columns for better visualization.


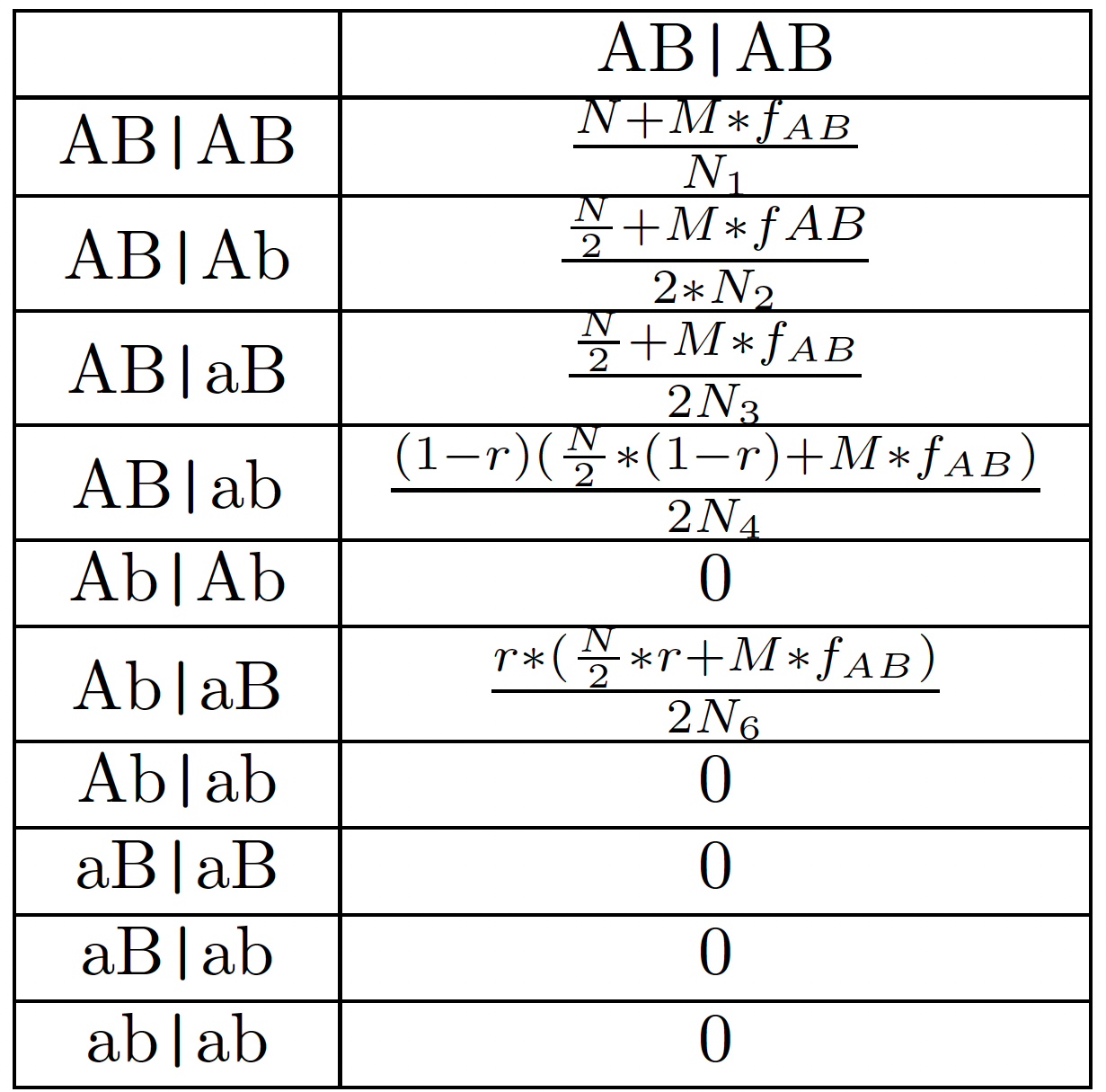


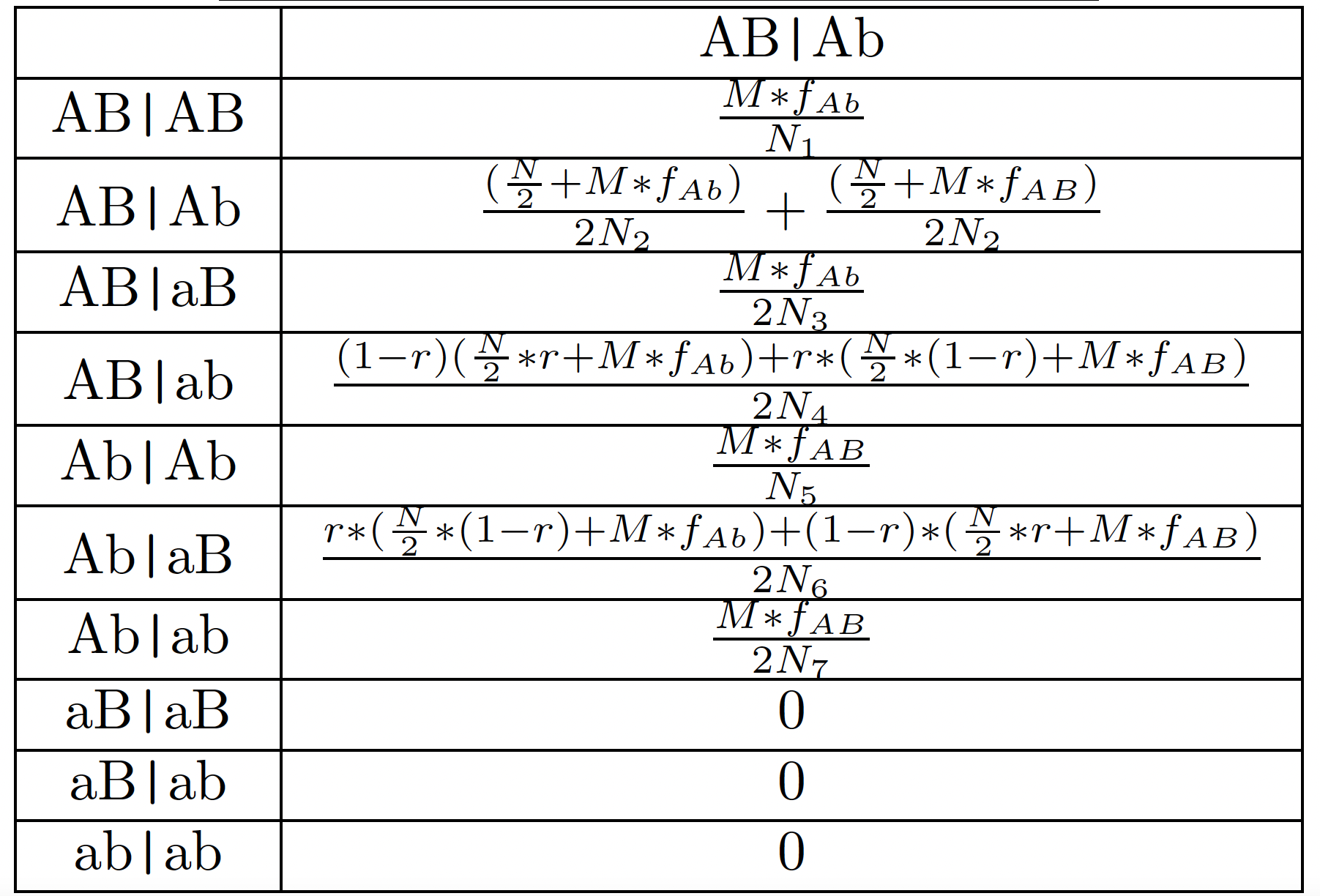


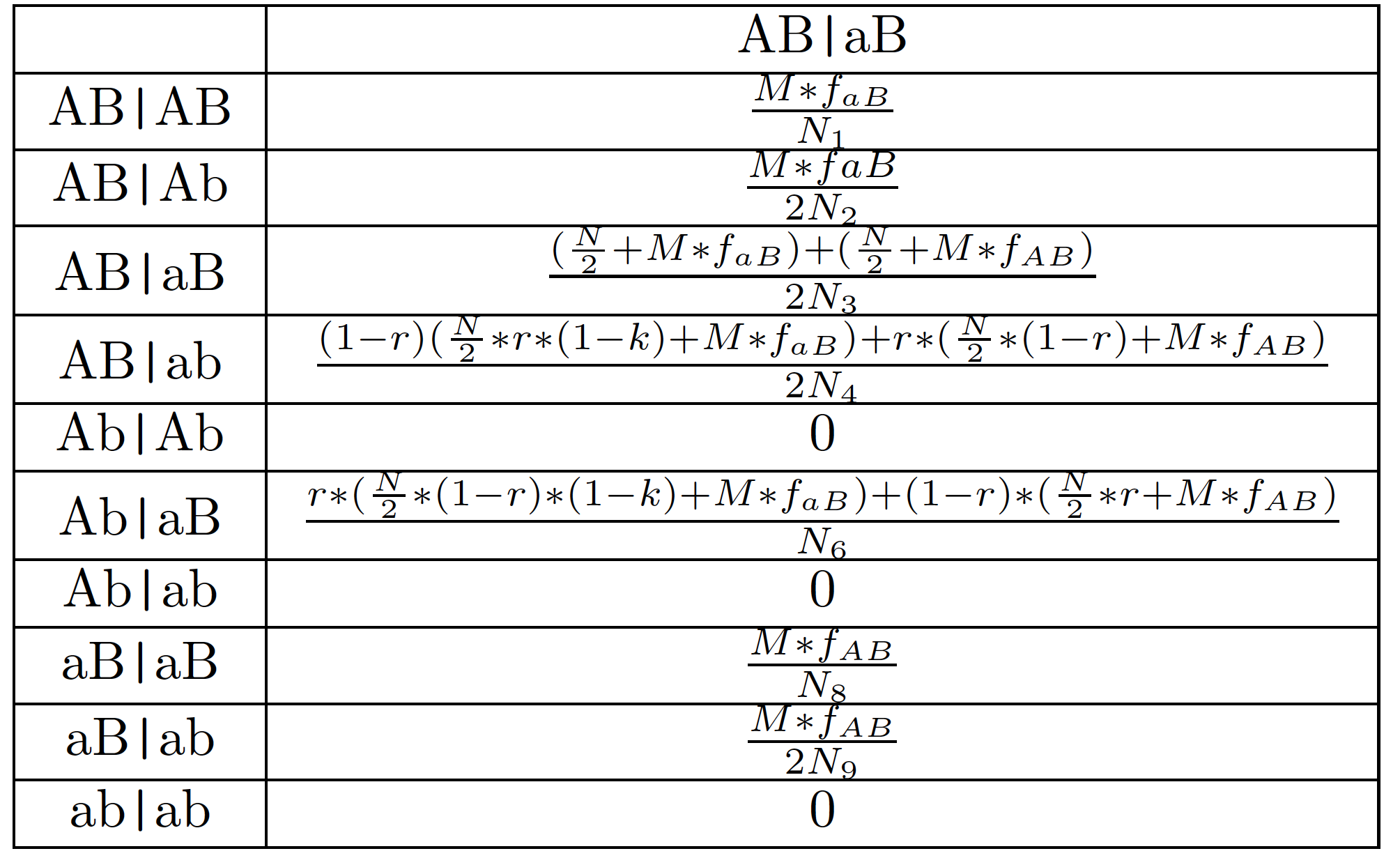


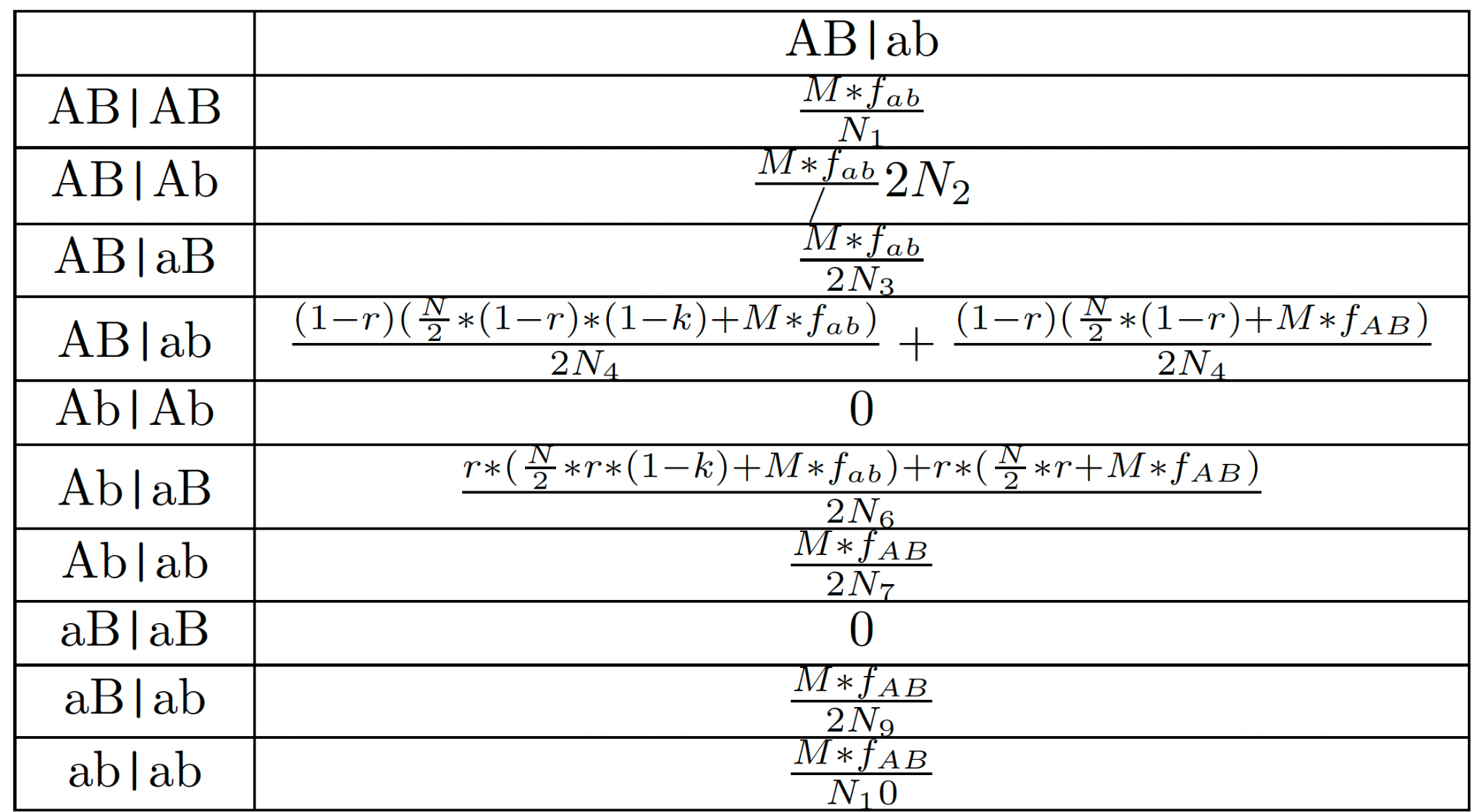


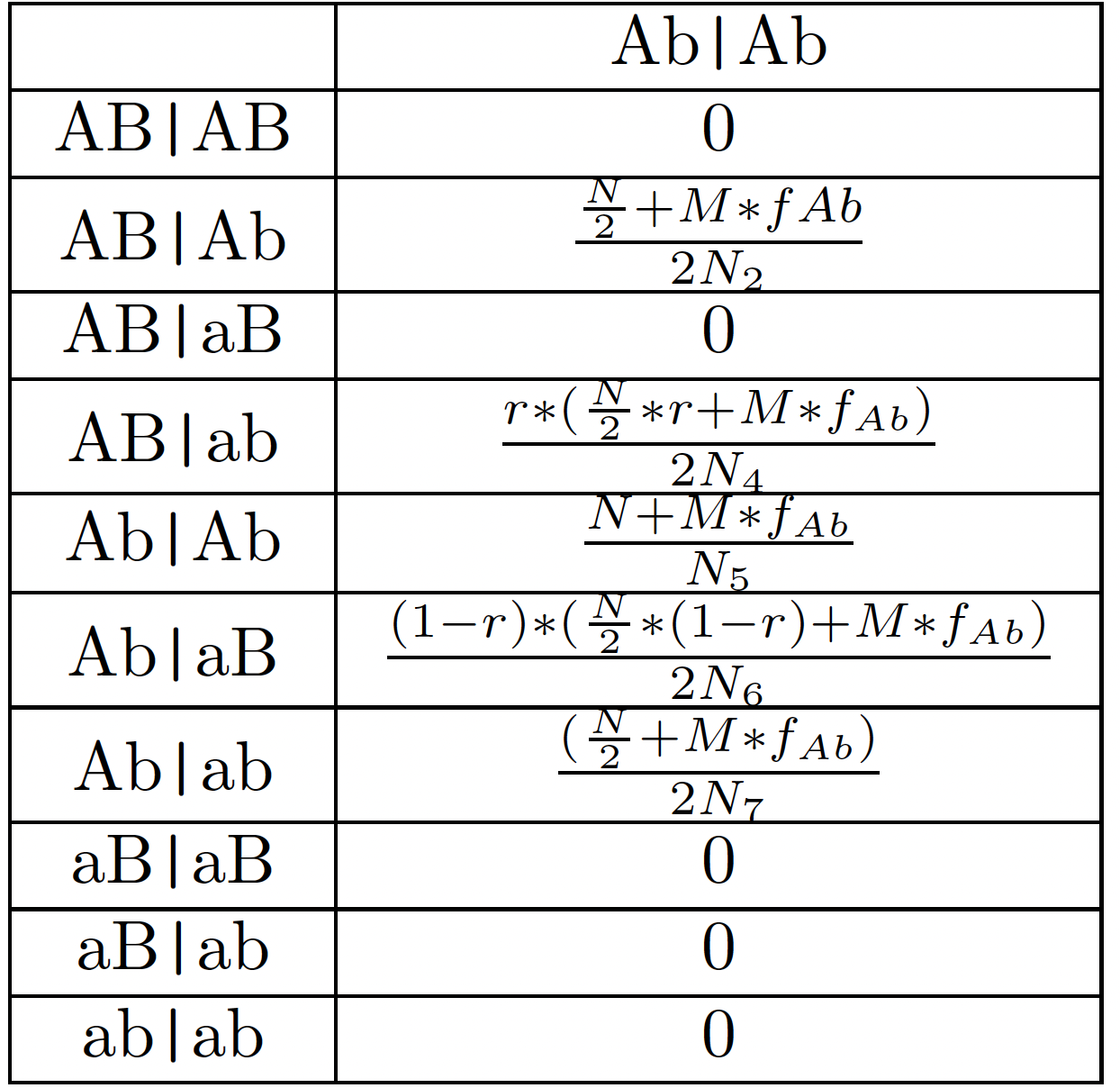


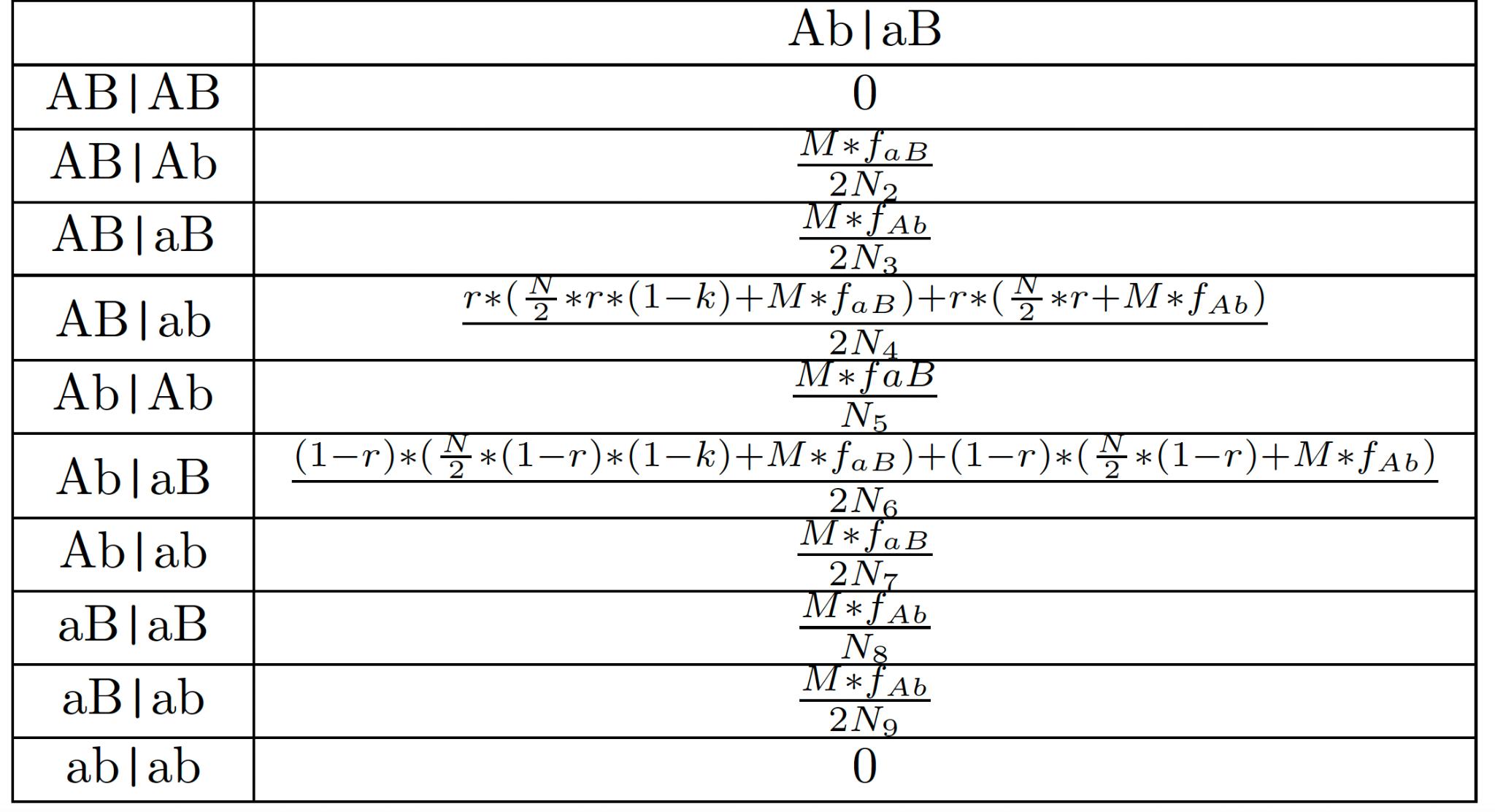


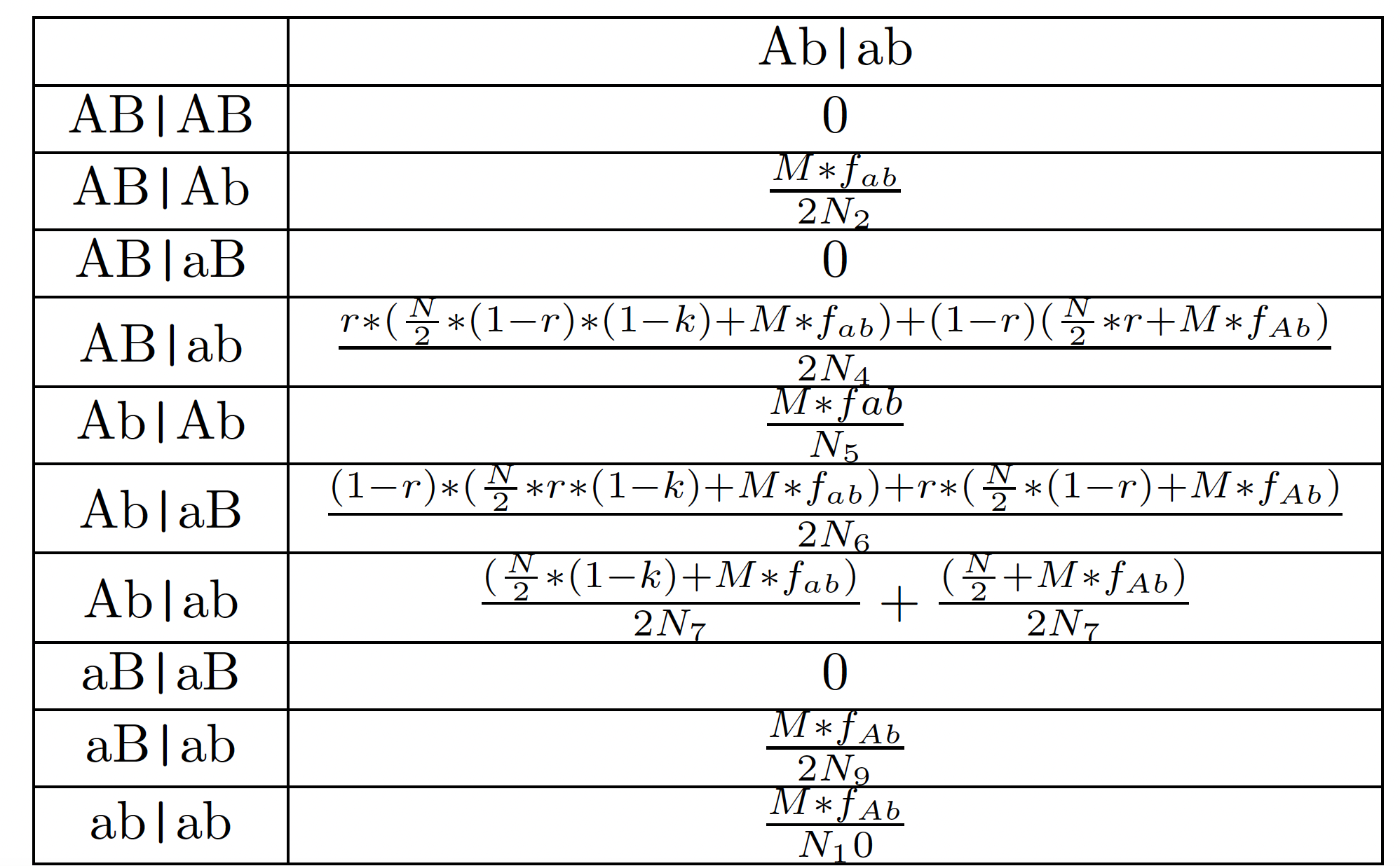


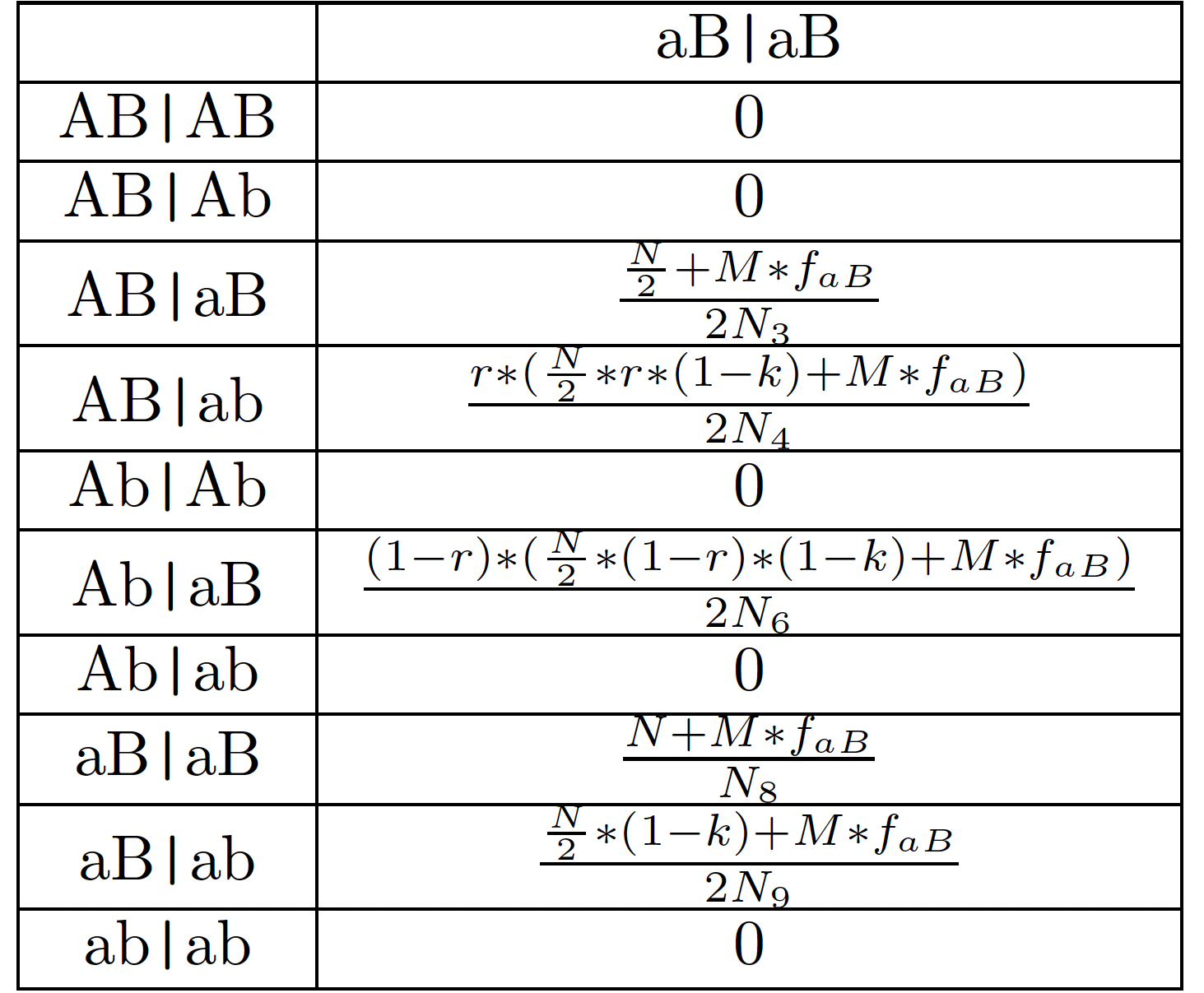


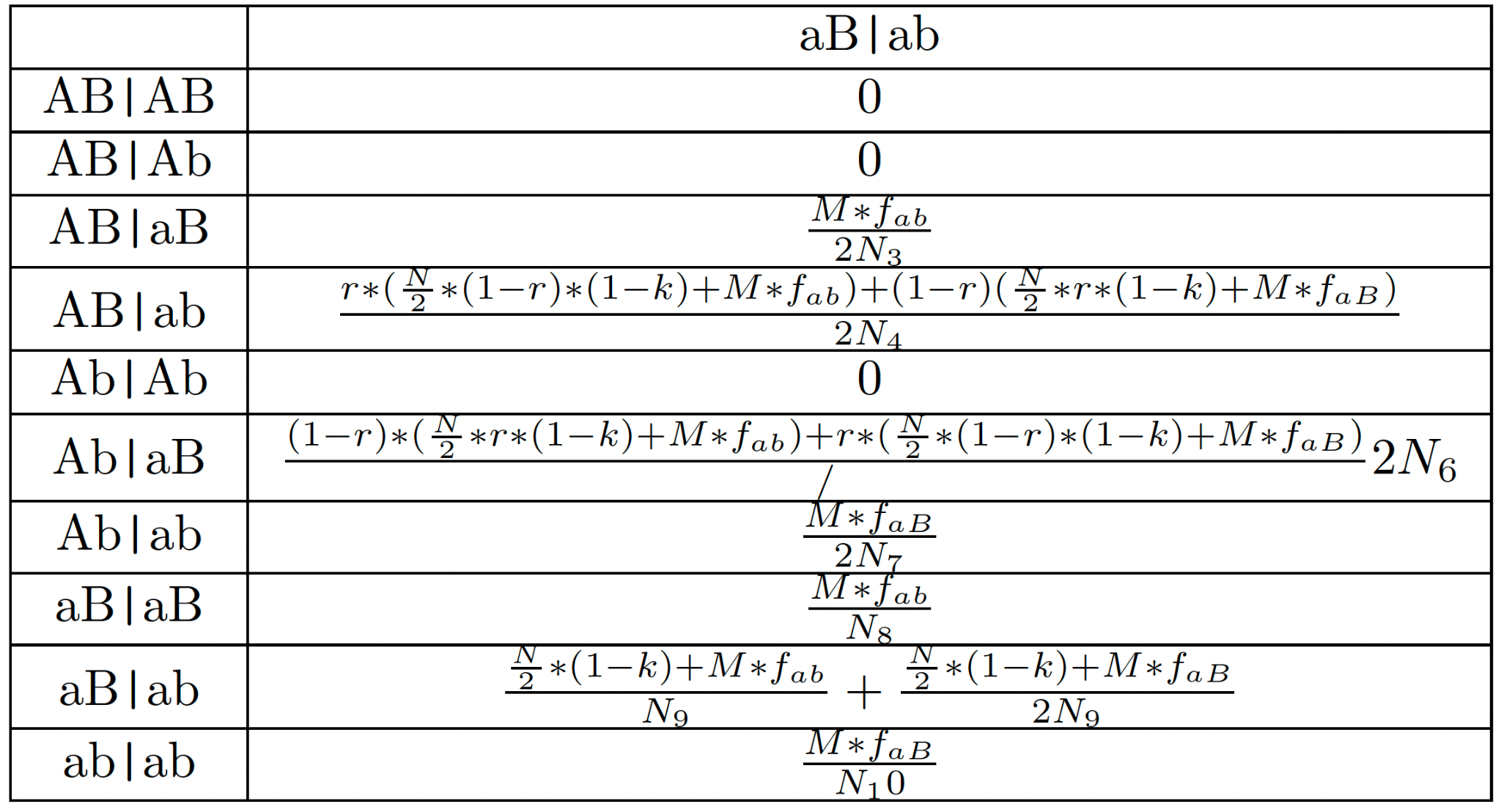


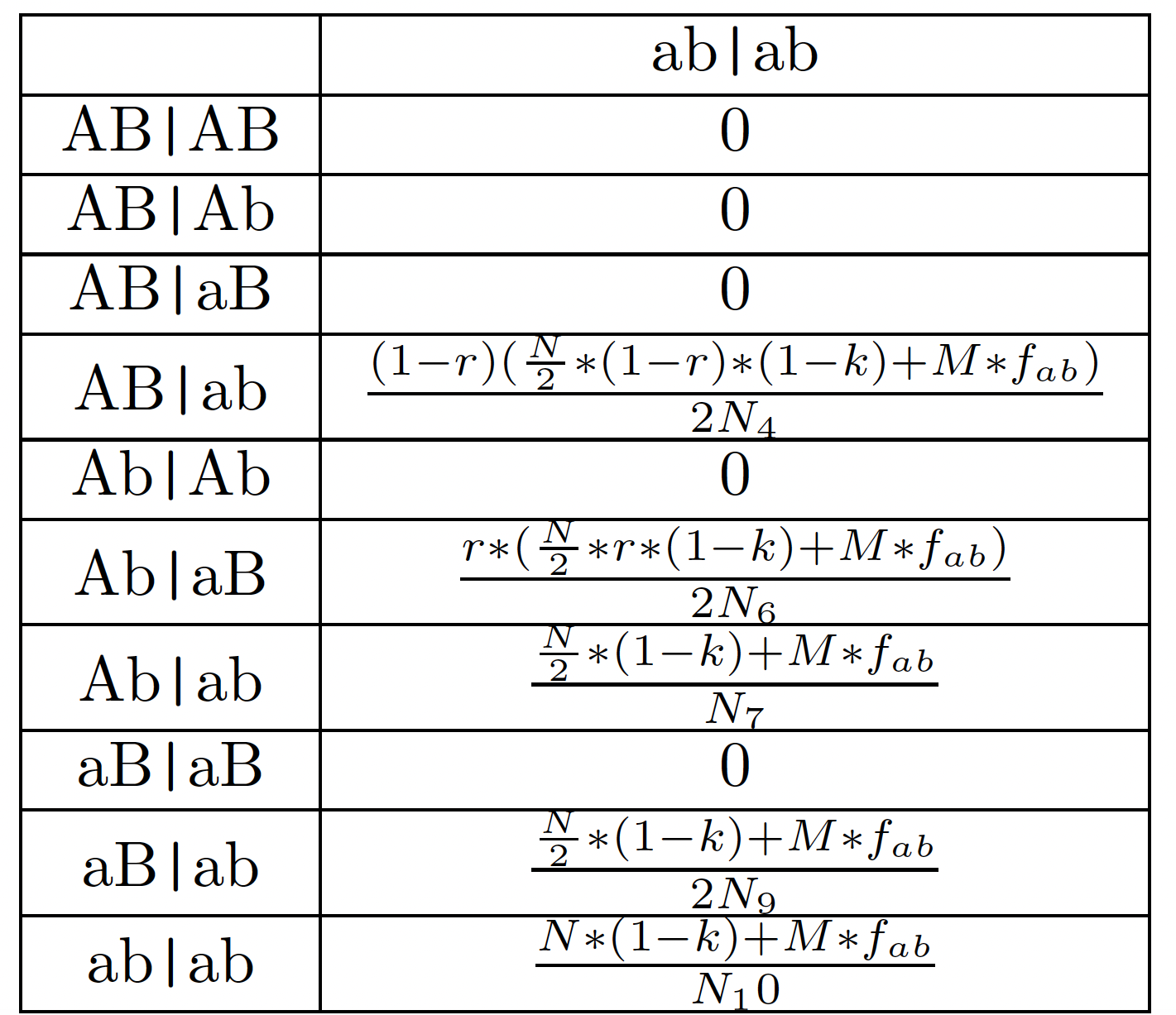


**Table S5**. The data source of high-quality genome assemblies used in this study.

| Species | Data source | Reference |
| --- | --- | --- |
| *Oryza sativa* ssp. *japonica* var. Nipponbare | <http://rice.uga.edu/>, version 7.0 | [(Kawahara et al., 2013)](https://paperpile.com/c/afK3iD/LkZhj) |
| *Oryza sativa* ssp. *indica* var. Minghui63 | NCBI: CP054676–CP054688 | [(Li et al., 2021)](https://paperpile.com/c/afK3iD/JdY0J) |
| *Oryza barthii* | NCBI: GCA_000182155.4 | [(Stein et al., 2018)](https://paperpile.com/c/afK3iD/hahnv) |
| *Oryza glaberrima* | NCBI: GCA_000147395.3 | [(Stein et al., 2018)](https://paperpile.com/c/afK3iD/hahnv) |
| *Oryza glumaepatula* | NCBI: GCA_000576495.2 | [(Stein et al., 2018)](https://paperpile.com/c/afK3iD/hahnv) |
| *Oryza longistaminata* | http://olinfres.nig.ac.jp/ | [(Reuscher et al., 2018)](https://paperpile.com/c/afK3iD/S1Blg) |
| *Oryza meridionalis* | NCBI: GCA_000338895.3 | [(Stein et al., 2018)](https://paperpile.com/c/afK3iD/hahnv) |

##

## Supplementary Figures


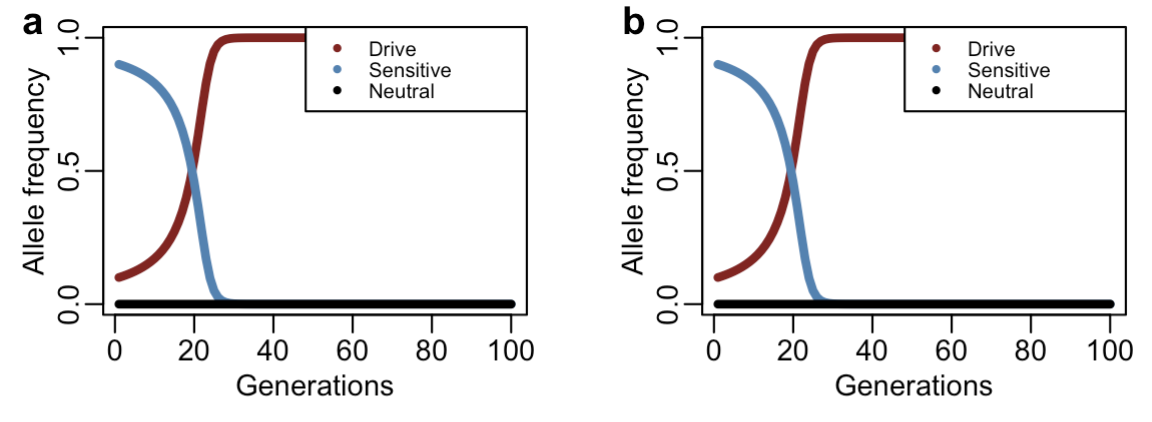


**Figure S1**. Expected allele frequency of different haplotypes as a function of time since the introduction of the SD haplotype. The calculations assume initial frequencies of 0.1 and 0.9 for the killer and sensitive haplotypes, respectively. The killing efficiency parameter, *k*, is set as 1. There is neither selfing nor recombination, the killer haplotype simply replaces the sensitive haplotype. The recombination rate, *r*, is set as 0 and the selfing rate, *s*, is set as 1e-5. The pollen redundancy level, *R*, is set as 1.5 in (**a**) and 1 in (**b**). These figures are to be contrasted with Fig. 1A.


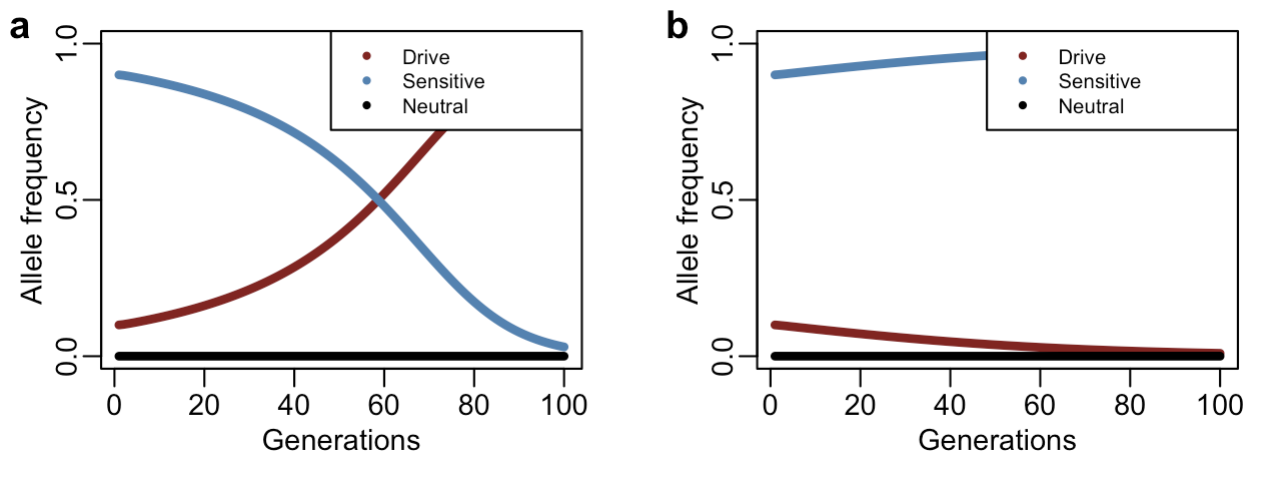


**Figure S2**. Expected allele frequency of different haplotypes as a function of time since the introduction of the SD haplotype. The calculations assume initial frequencies of 0.1 and 0.9 for the killer and sensitive haplotypes, respectively. The killing efficiency parameter, *k*, is set as 1. The recombination rate, *r*, is set as 0 and the selfing rate, *s*, is set as 0.9. The pollen redundancy level, *R*, is set as 1.5 in (**a**) and 1 in (**b**). These figures are to be contrasted with Fig. 1B.


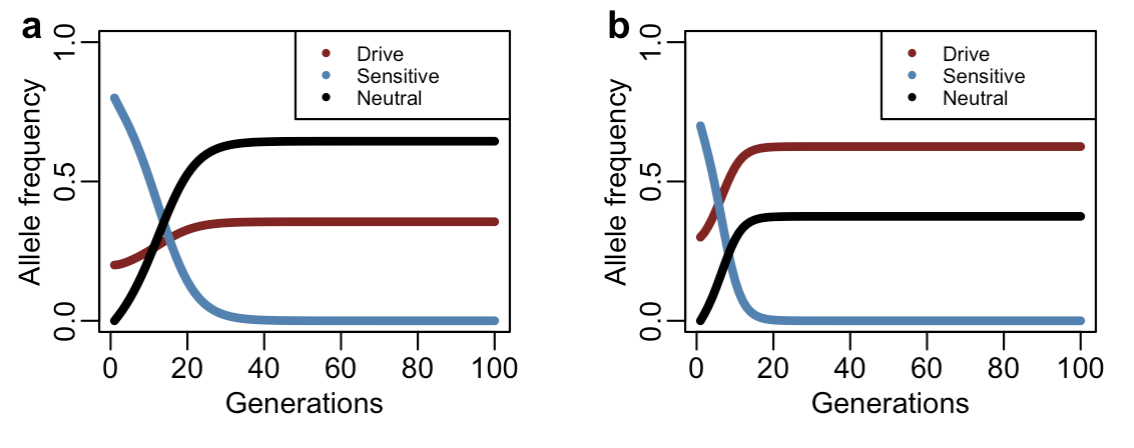


**Figure S3**. Expected allele frequency of different haplotypes as a function of time since the introduction of the SD haplotype. The killing efficiency parameter, *k*, is set as 1. The recombination rate, *r*, is set as 0.1 and the selfing rate, *s*, is set as 1e-5. The pollen redundancy level, *R*, is set as 10. (**A**) The initial frequencies of 0.2 and 0.8 for the killer and sensitive haplotypes, and (**B**) the initial frequencies of 0.3 and 0.7 for the killer and sensitive haplotypes. These figures are to be contrasted with Fig. 1C.


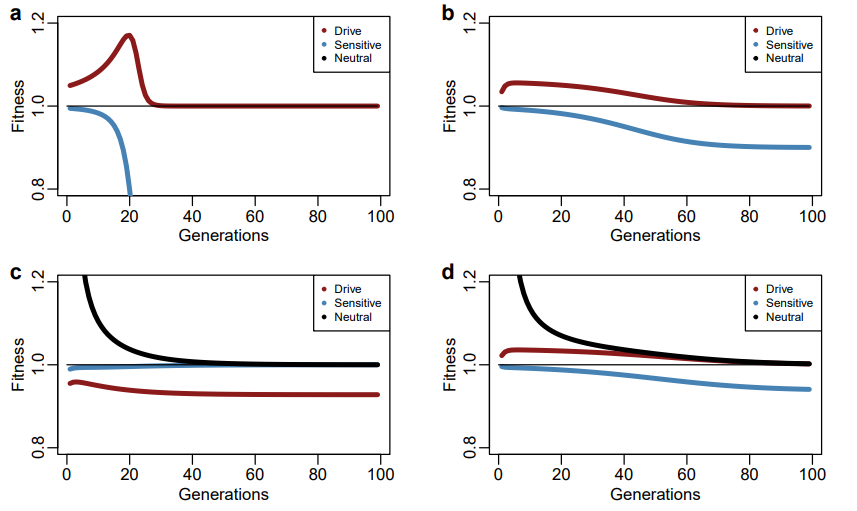


**Figure S4**. Expected fitness of different haplotypes as a function of time since the introduction of the SD haplotype. The calculations assume initial frequencies of 0.1 and 0.9 for the killer and sensitive haplotypes, respectively. The pollen redundancy level, *R*, is set as 10. The killing efficiency parameter, *k*, is set as 1. In (**a**), the simplest case, there is neither selfing nor recombination, the killer haplotype simply replaces the sensitive haplotype. The recombination rate, *r*, is set as 0 and the selfing rate, *s*, is set as 1e-5. In (**b**), selfing (*s* = 0.9) is added; (**c**) recombination is added (*r* = 0.1) and (**d**) both recombination (*r* = 0.1) and selfing (*s* = 0.9) are added.


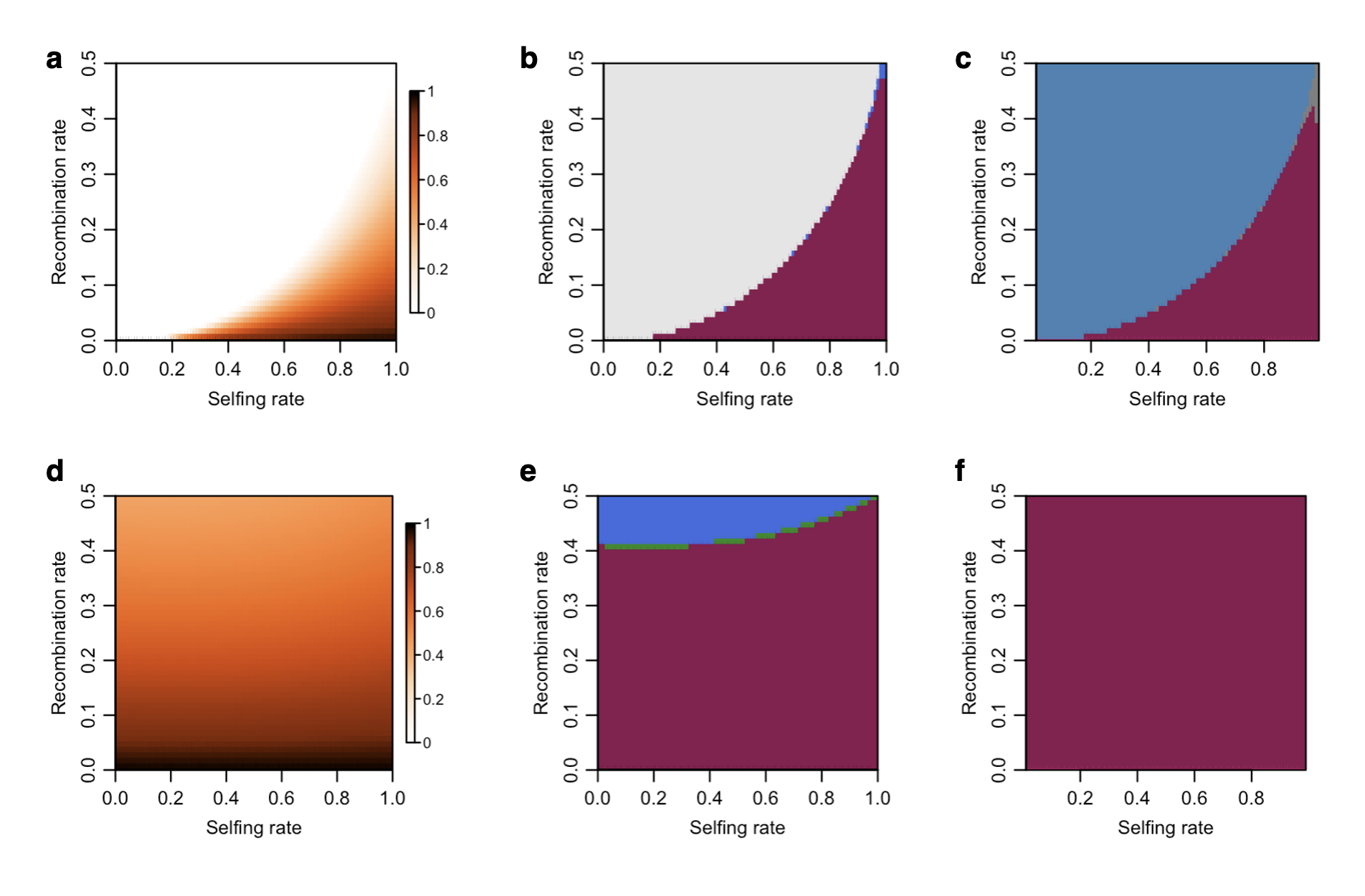


**Figure S5**. Selfing rate, recombination rate and the dynamics of haplotype frequencies. The calculations were run with a maximum of 10000 generations until the proportion of either Ab or aB became less than 0.001. (**a-c**) In the initial population, the frequency of sensitive haplotype is 0.999, and killer haplotype is 0.001. (**d-e**) Frequencies of both the killer and sensitive haplotypes are 0.5. (**a.d**) the color shows the allele frequency of the killer haplotype. (**b.e**) The color shows the state of the killer haplotype. Dark red, increased in frequency; blue, decreased in frequency; gray, removed from population. (**c.f**) The color shows the state of the population. Dark red, killer and neutral haplotypes coexist; blue, sensitive and neutral haplotypes coexist; gray, killer, sensitive and neutral haplotypes coexist; magenta, only killer haplotype exists.


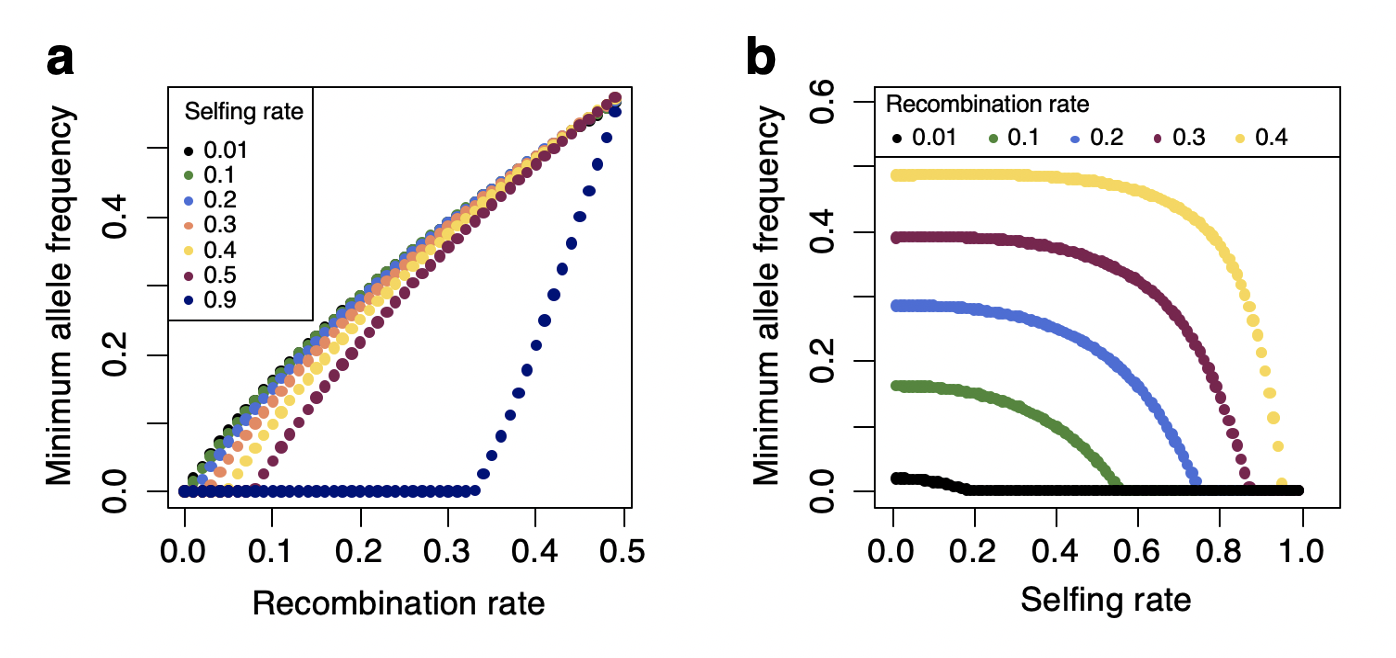


**Figure S6**. Minimum initial frequency, recombination rates, selfing rates, and spread of killer haplotype. The modeling is based on invasion of populations with sensitive haplotype by the killer haplotype. The killer was determined to be able to spread if the allele frequency after 100 generations is greater than the initial frequency. (**a**) Relationship between minimum initial frequency and recombination rate with varying selfing rates. (**b**) Relationship between minimum initial frequency and selfing rate with varying recombination rates.


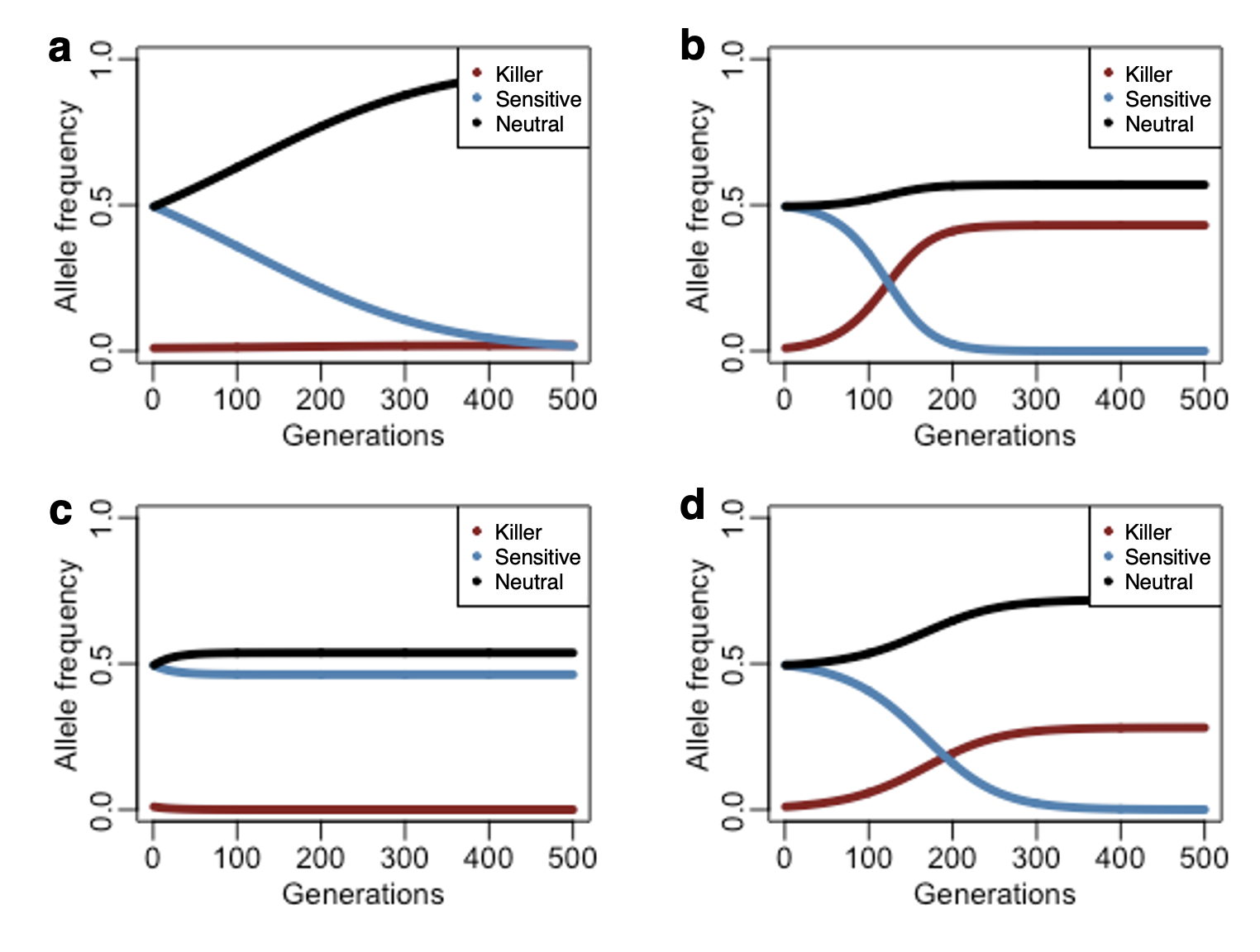


**Figure S7**. Expected allele frequency of different haplotypes as a function of time since the introduction of the SD haplotype. In all cases the killing efficiency parameter *k* is set to 1.The parameters *r*, *k* and *s* are the same as in Fig. 1. The killer haplotype arises as a new mutation with the initial haplotype frequency set as 0.001, and initial frequencies of neutral and sensitive haplotypes are 0.4995 respectively. In (**a**), the simplest case, there is neither selfing nor recombination, the killer haplotype replaces the sensitive one. (**c**) selfing (*s* = 0.9) is added; (**d**) recombination is added (*r* = 0.1) and (**d**) both recombination (*r* = 0.1) and selfing (*s* = 0.9) are added.


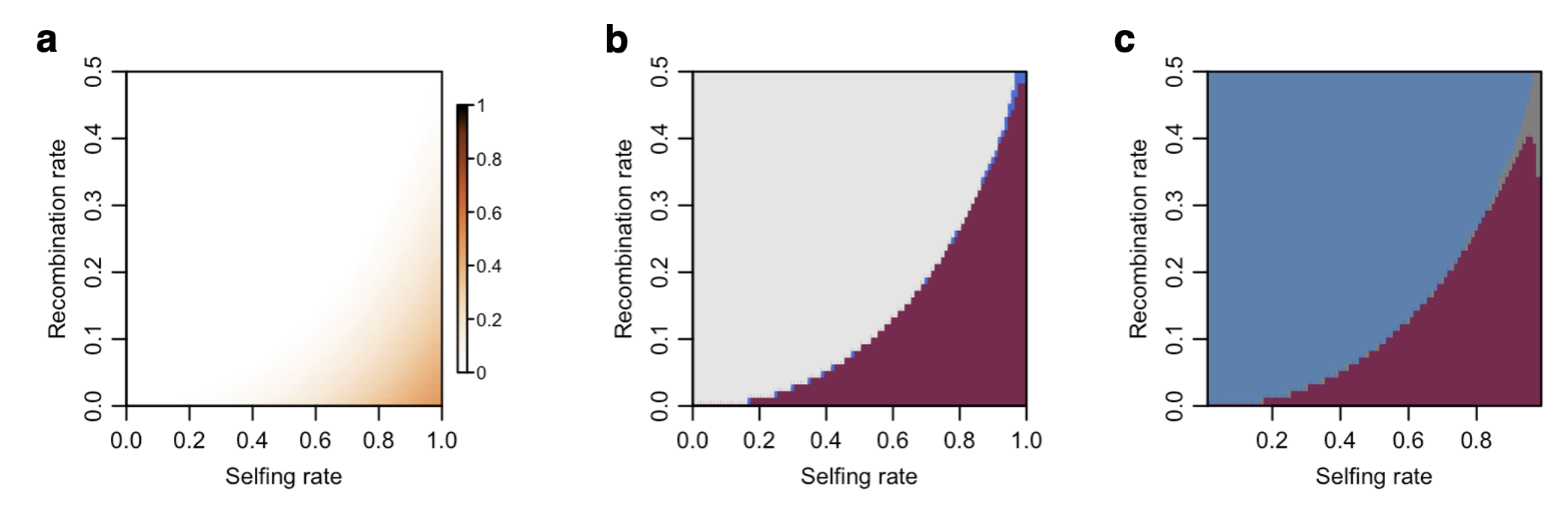


**Figure S8**. invasion with presence of neutral allele varying selfing and recombination rates. The calculations were run with a maximum of 10000 generations until the proportion of either Ab or aB became less than 0.001. In the initial population, the frequency of sensitive and neutral haplotype is 0.4995, and killer haplotype is 0.001. (**a**) The color shows the allele frequency of the killer haplotype. (**b**) The color shows the state of the killer haplotype after 10000 generations. Dark red, increased in frequency; blue, decreased in frequency; gray, removed from population. (**c**) The color shows the state of the population after 10000 generations. Dark red, killer and neutral haplotypes coexist; blue, sensitive and neutral haplotypes coexist.


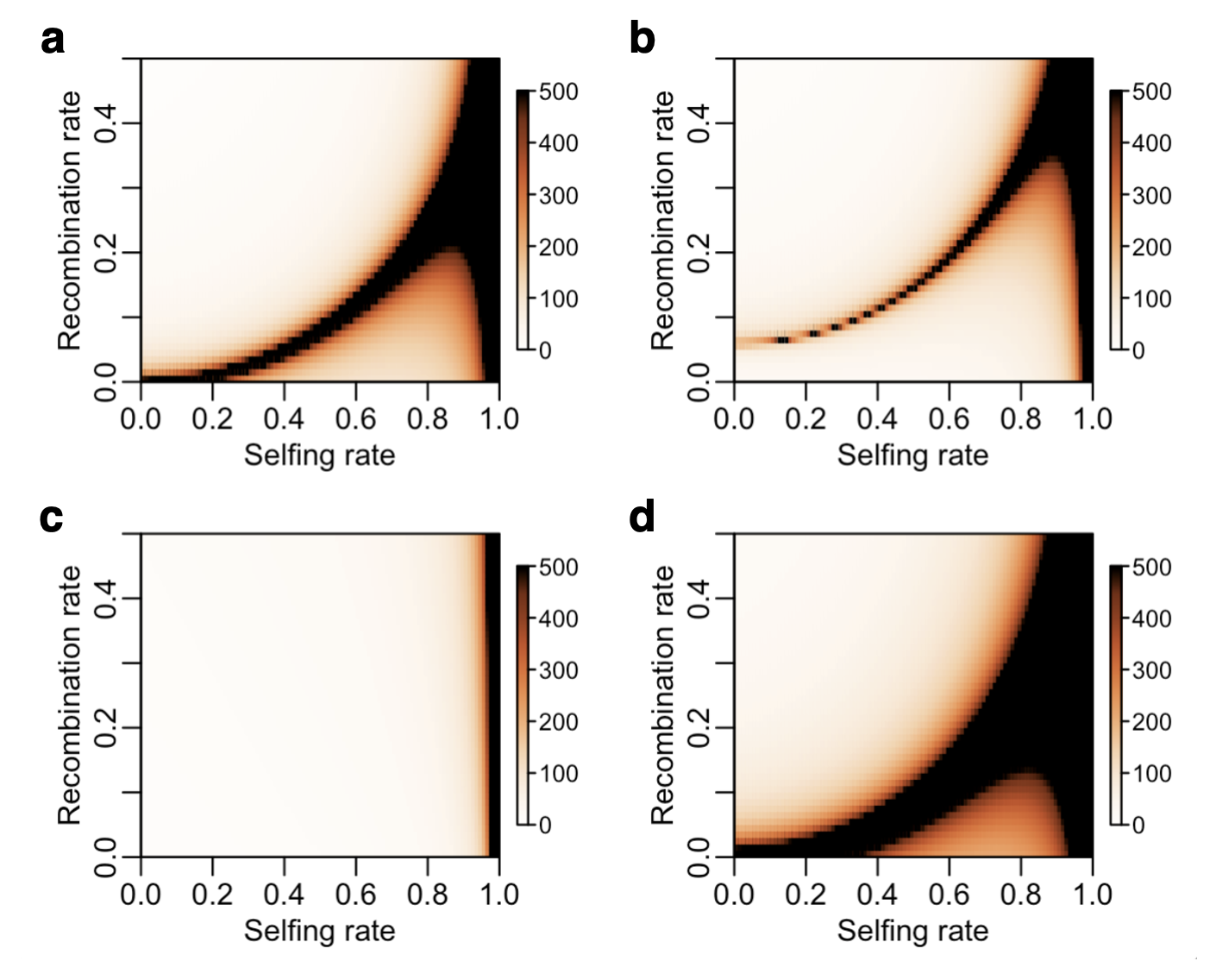


**Figure S9**. Selfing rate, recombination rate and the generations before equilibria. The color indicates the number of generations before reaching equilibria, and it is capped at 500 generations (numbers greater than 500 are all denoted as black). The calculations were run for a maximum of 10000 generations with pollen redundancy level *R* set as 10. (**a**)The initial frequency of the killer haplotype is 0.001. (**b**) The initial frequency of the killer haplotype is 0.1. (**c**) The initial frequency of the killer haplotype is 0.5. (**d**) The killer haplotype arises as a new mutation, the initial frequency of haplotype is 0.001, and initial frequencies of neutral and sensitive haplotypes are 0.4995 respectively.


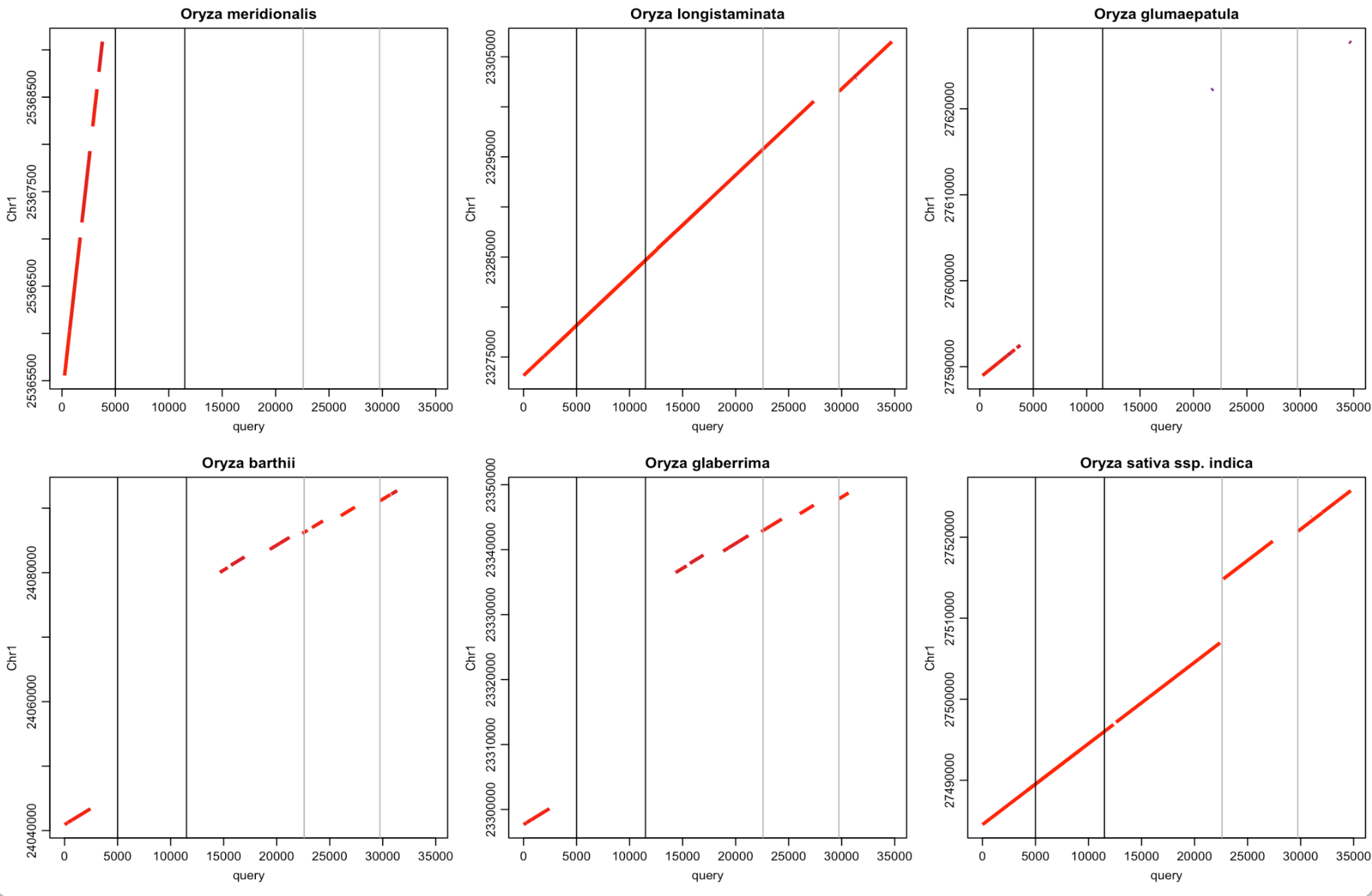


**Figure S10**. Synteny analysis at the *qHMS1* locus. The query is the ~35kb sequence at the *qHMS1* locus from the *O. sativa* ssp. *japonica* (Nipponbare) genome. The black lines delimit the *ORF3* and the gray lines delimit the *ORF5*.


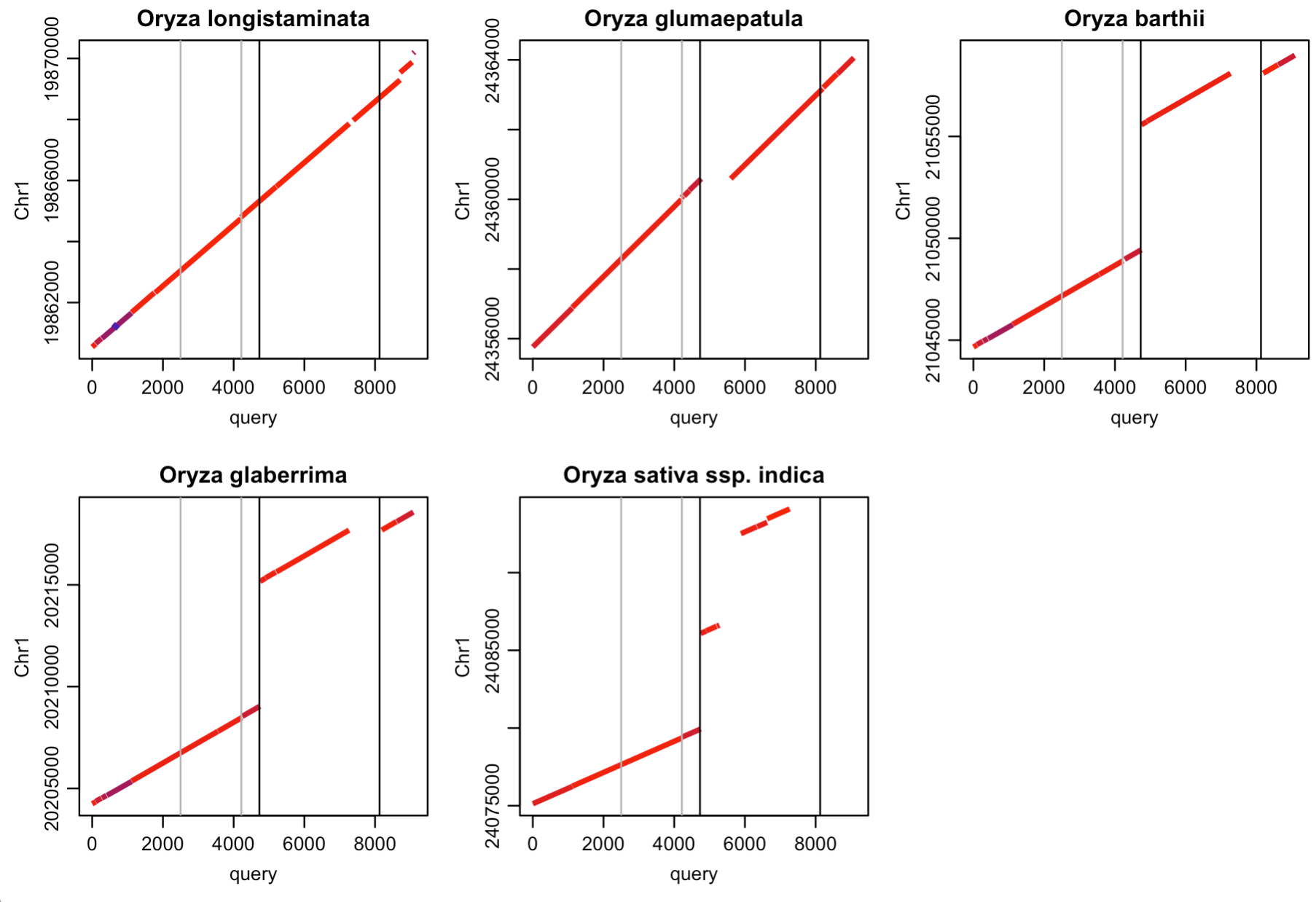


**Figure S11**. Synteny analysis at the *Sa* locus. The query is the ~9kb sequence at the *Sa* locus from the *O. sativa* ssp. *japonica* (Nipponbare) genome. The gray lines delimit the *SaF* gene and the black lines delimit the *SaM* gene.


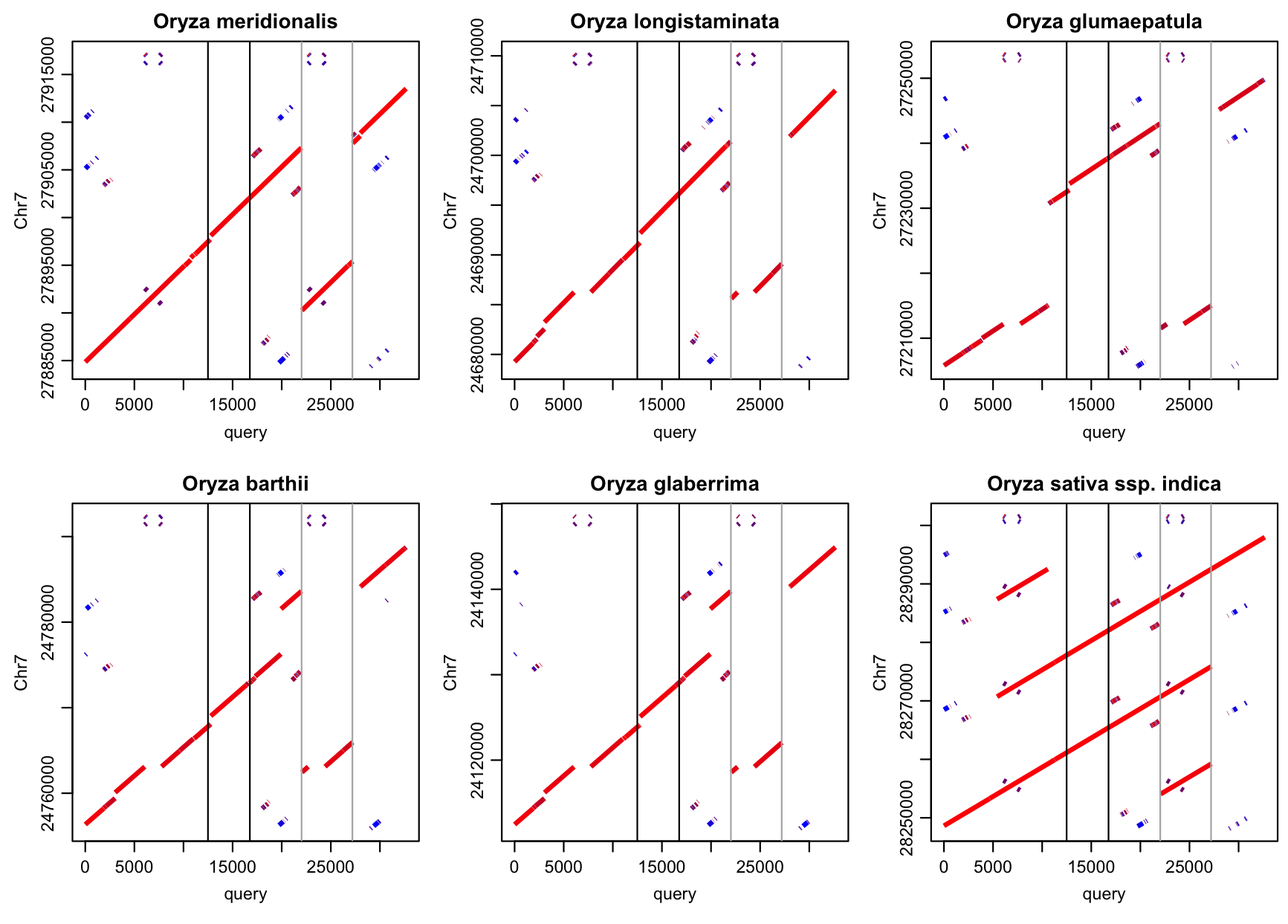


**Figure S12**. Synteny analysis at the *qHMS7* locus. The query is the ~30-kb sequence at the *qHMS7* locus from the *O. sativa* ssp. *japonica* (Nipponbare) genome. The black lines delimit the *ORF2* gene and the gray lines delimit the *ORF3* gene.


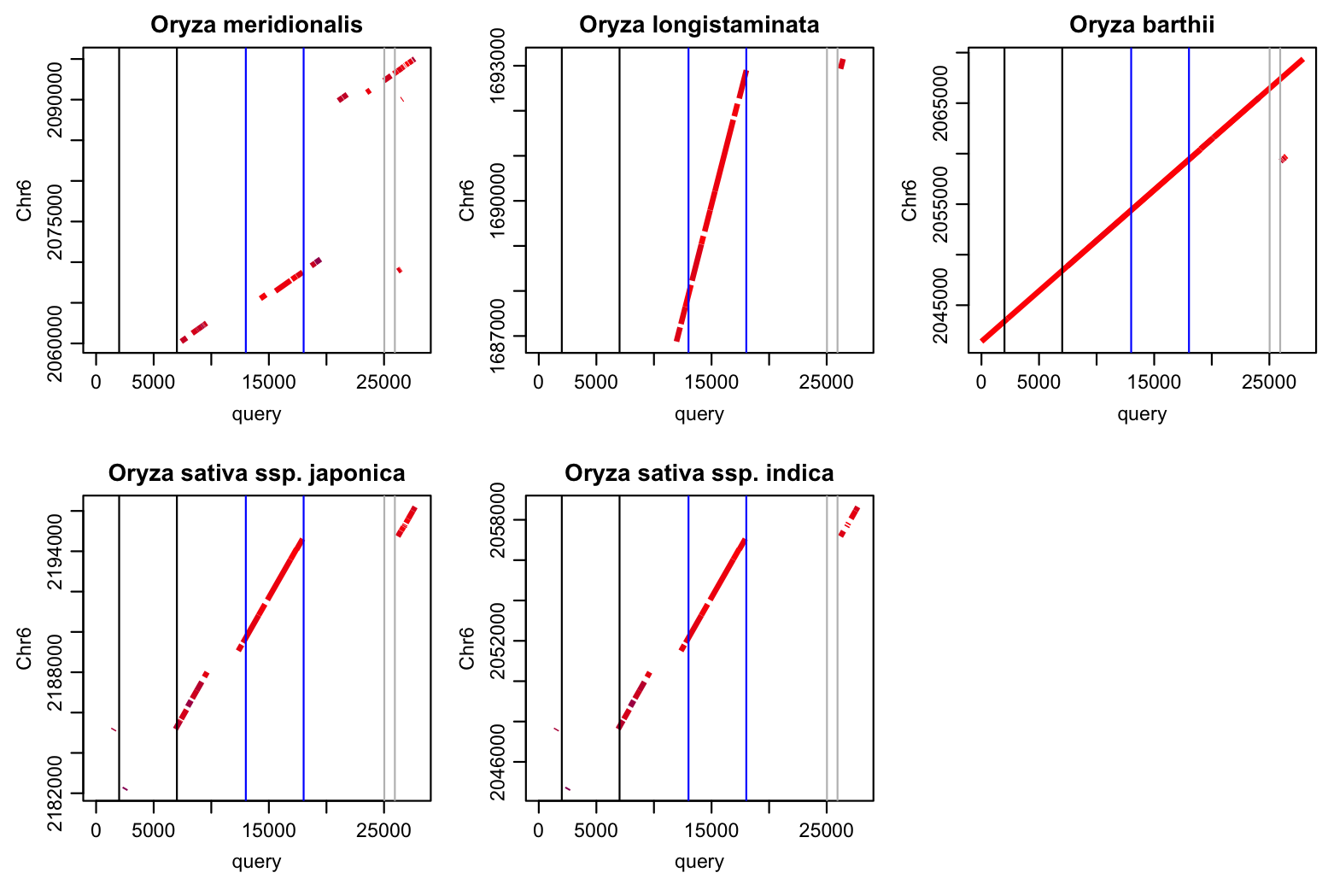


**Figure S13**. Synteny analysis at the *S1* locus. The query is the ~30-kb sequence at the *S1* locus from the *O. glaberrima* (GCA_000576495.2) genome. The black lines delimit the *S1A4* gene, the gray lines delimit the *S1A6* gene, and the blue lines delimit the *S1TPR* gene.


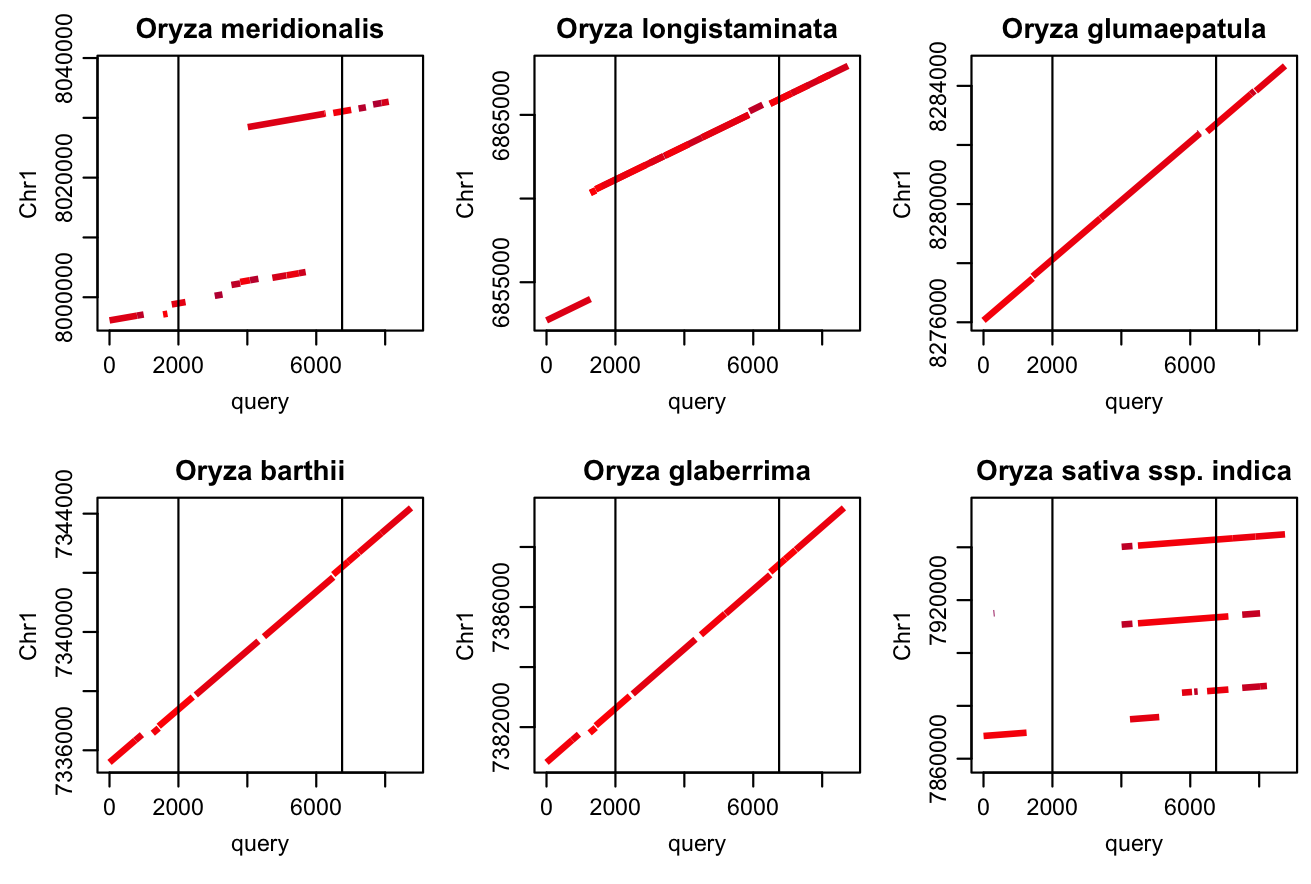


**Figure S14**. Synteny analysis at the *Sc* locus. The query is the ~8-kb sequence at the *Sc* locus from the *O. sativa* ssp. *japonica* (Nipponbare) genome. The black lines delimit the DUF1618-coding gene in the region.

##

## References

[**Kawahara, Y. et al.** (2013). Improvement of the Oryza sativa Nipponbare reference genome using next generation sequence and optical map data. Rice **6**: 4.](http://paperpile.com/b/afK3iD/LkZhj)

[**Koide, Y. et al.** (2018). Lineage-specific gene acquisition or loss is involved in interspecific hybrid sterility in rice. Proc. Natl. Acad. Sci. U. S. A. **115**: E1955–E1962.](http://paperpile.com/b/afK3iD/npzN7)

[**Li, K., Jiang, W., Hui, Y., Kong, M., Feng, L.-Y., Gao, L.-Z., Li, P., and Lu, S.** (2021). Gapless indica rice genome reveals synergistic contributions of active transposable elements and segmental duplications to rice genome evolution. Mol. Plant **14**: 1745–1756.](http://paperpile.com/b/afK3iD/JdY0J)

[**Long, Y. et al.** (2008). Hybrid male sterility in rice controlled by interaction between divergent alleles of two adjacent genes. Proc. Natl. Acad. Sci. U. S. A. **105**: 18871–18876.](http://paperpile.com/b/afK3iD/wnyxj)

[**Reuscher, S., Furuta, T., Bessho-Uehara, K., Cosi, M., Jena, K.K., Toyoda, A., Fujiyama, A., Kurata, N., and Ashikari, M.** (2018). Assembling the genome of the African wild rice Oryza longistaminata by exploiting synteny in closely related Oryza species. Commun Biol **1**: 162.](http://paperpile.com/b/afK3iD/S1Blg)

[**Shen, R., Wang, L., Liu, X., Wu, J., Jin, W., Zhao, X., Xie, X., Zhu, Q., Tang, H., Li, Q., Chen, L., and Liu, Y.-G.** (2017). Genomic structural variation-mediated allelic suppression causes hybrid male sterility in rice. Nat. Commun. **8**: 1310.](http://paperpile.com/b/afK3iD/yMqTu)

[**Stein, J.C. et al.** (2018). Genomes of 13 domesticated and wild rice relatives highlight genetic conservation, turnover and innovation across the genus Oryza. Nat. Genet. **50**: 285–296.](http://paperpile.com/b/afK3iD/hahnv)

[**Wang, C. et al.** (2023a). A natural gene drive system confers reproductive isolation in rice. Cell.](http://paperpile.com/b/afK3iD/s8piA)

[**Wang, D. et al.** (2023b). Two complementary genes in a presence-absence variation contribute to indica-japonica reproductive isolation in rice. Nat. Commun. **14**: 4531.](http://paperpile.com/b/afK3iD/Q49Dj)

[**Xie, Y. et al.** (2019). An asymmetric allelic interaction drives allele transmission bias in interspecific rice hybrids. Nat. Commun. **10**: 2501.](http://paperpile.com/b/afK3iD/REBDJ)

[**Xie, Y., Xu, P., Huang, J., Ma, S., Xie, X., Tao, D., Chen, L., and Liu, Y.-G.** (2017). Interspecific Hybrid Sterility in Rice Is Mediated by OgTPR1 at the S1 Locus Encoding a Peptidase-like Protein. Mol. Plant **10**: 1137–1140.](http://paperpile.com/b/afK3iD/oxvgK)

[**You, S. et al.** (2023). A toxin-antidote system contributes to interspecific reproductive isolation in rice. Nat. Commun. **14**: 7528.](http://paperpile.com/b/afK3iD/CBTZ)

[**Yu, X. et al.** (2018). A selfish genetic element confers non-Mendelian inheritance in rice. Science **360**: 1130–1132.](http://paperpile.com/b/afK3iD/PnVqZ)

[**Zhou, P. et al.** (2023). A minimal genome design to maximally guarantee fertile inter-subspecific hybrid rice. Mol. Plant **16**: 726–738.](http://paperpile.com/b/afK3iD/qnUwN)
